# Supplementary material for: Chk1 Inhibition of the Replication Factor Drf1 Guarantees Cell-Cycle Elongation at the Xenopus laevis Mid-blastula Transition
Source: Dev Cell. 2017 Jul 10;42(1):82–96.e3. doi: 10.1016/j.devcel.2017.06.010 (PMC5505860; doi:10.1016/j.devcel.2017.06.010)
Supplement: Document S1. Figures S1–S7 [file mmc1.pdf]

Developmental Cell, Volume *42*

Supplemental Information

Chk1 Inhibition of the Replication Factor Drf1 Guarantees Cell-Cycle Elongation at the *Xenopus laevis* Mid-blastula Transition

Clara Collart, James C. Smith, and Philip Zegerman

Figure S1

A. Chk1 D148A overexpression inhibits phosphorylation of a Chk1 target

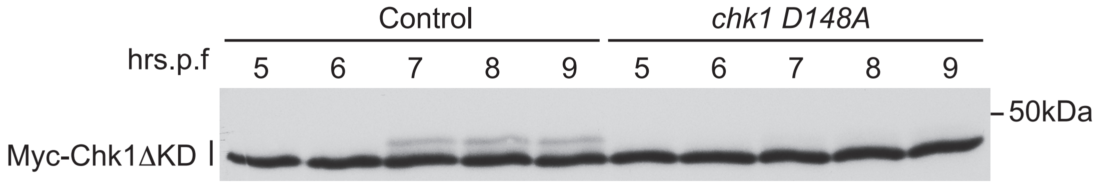

B. Chk1 D148A over-expression alone does not cause an increase in DNA content after the MBT

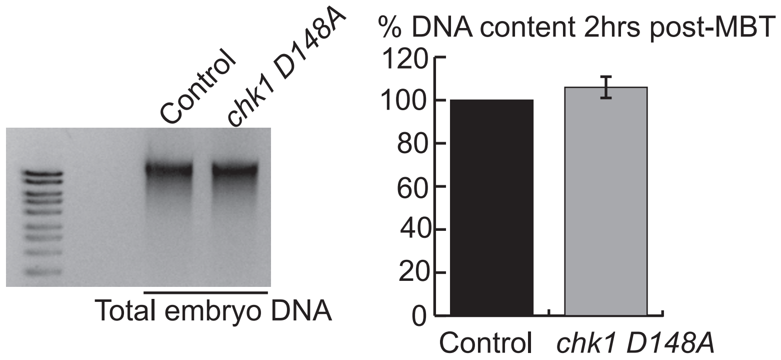

C. Wild type Chk1 over-expression causes premature Chk1 activation

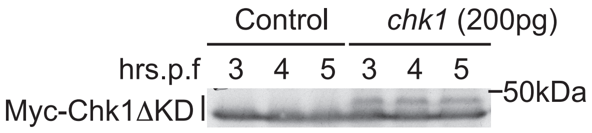

D. Chk1 is a potent inhibitor of cell cycle progression in the early embryo

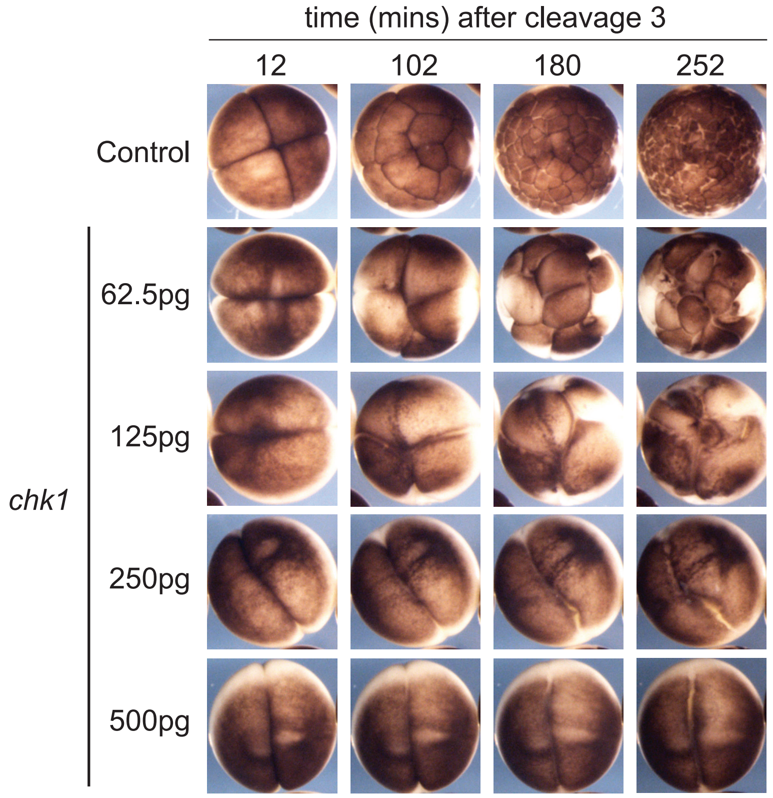

E. Drf1 or Dbf4 over-expression does not affect Chk1 activation

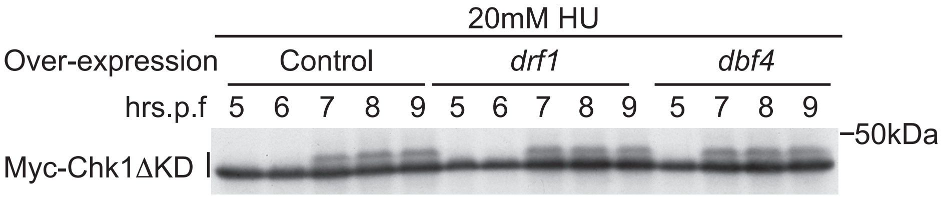

Figure S2.
Only over-expression of *treslin*, *recq4* and *cut5* together with the *chk1 D148A* mutant permits fast MBT cycles

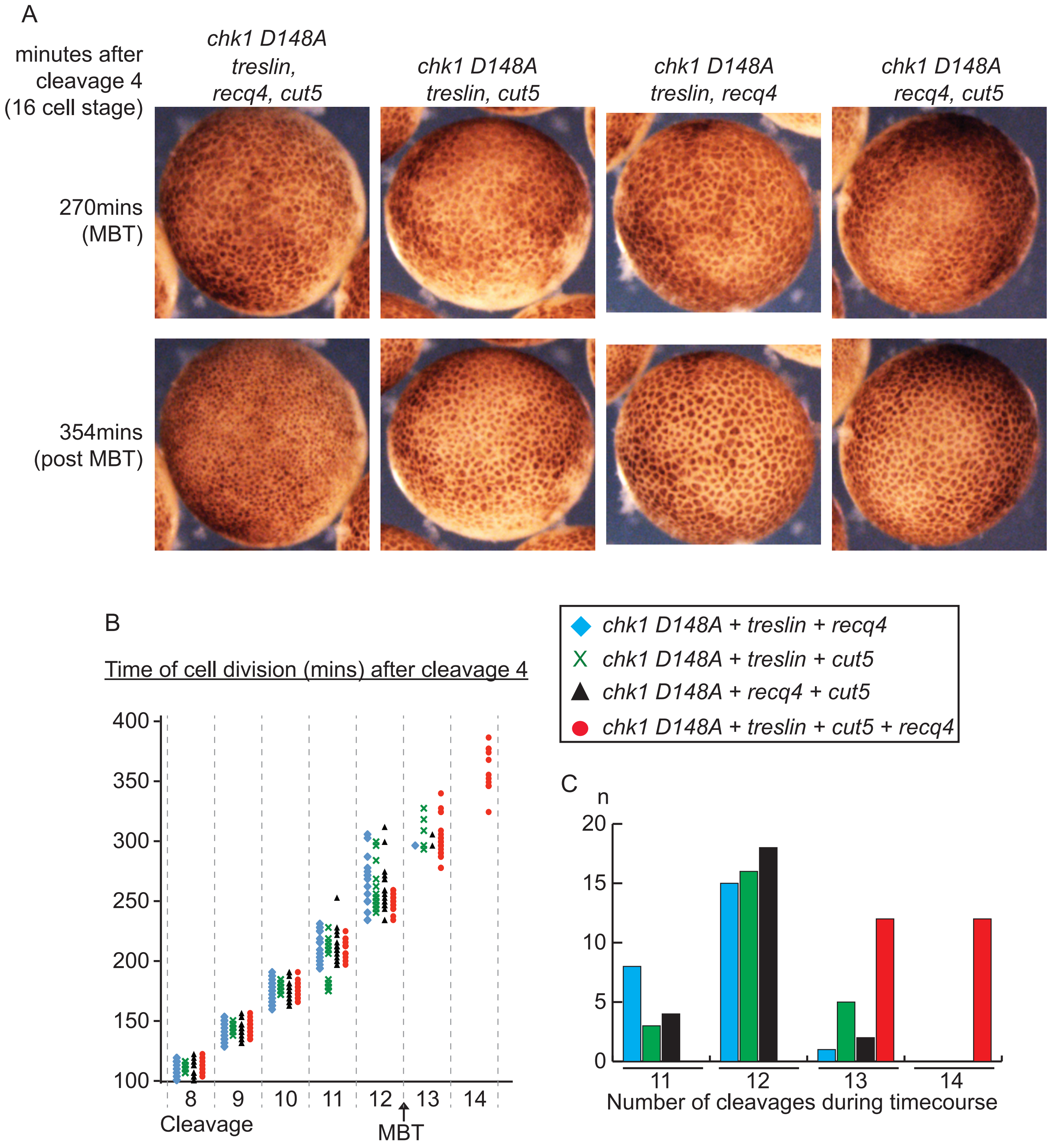

Figure S3.
Inhibition of DNA replication by injection of aphidicolin is sufficient to block the cell division, pre-MBT.

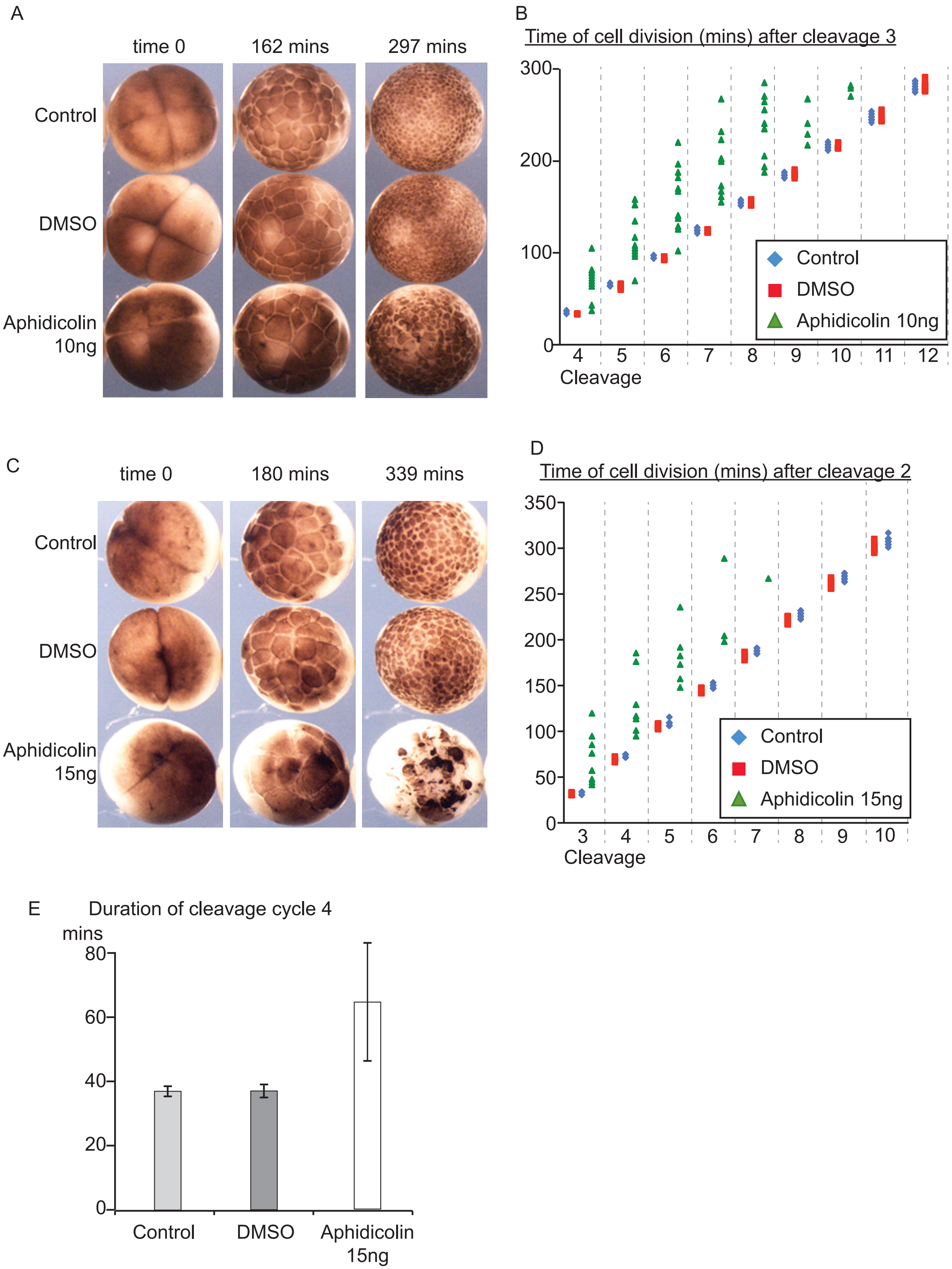

Figure S4.
Over-expression of *cdk1-AF* does not prevent Chk1-mediated elongation of pre-MBT cycles

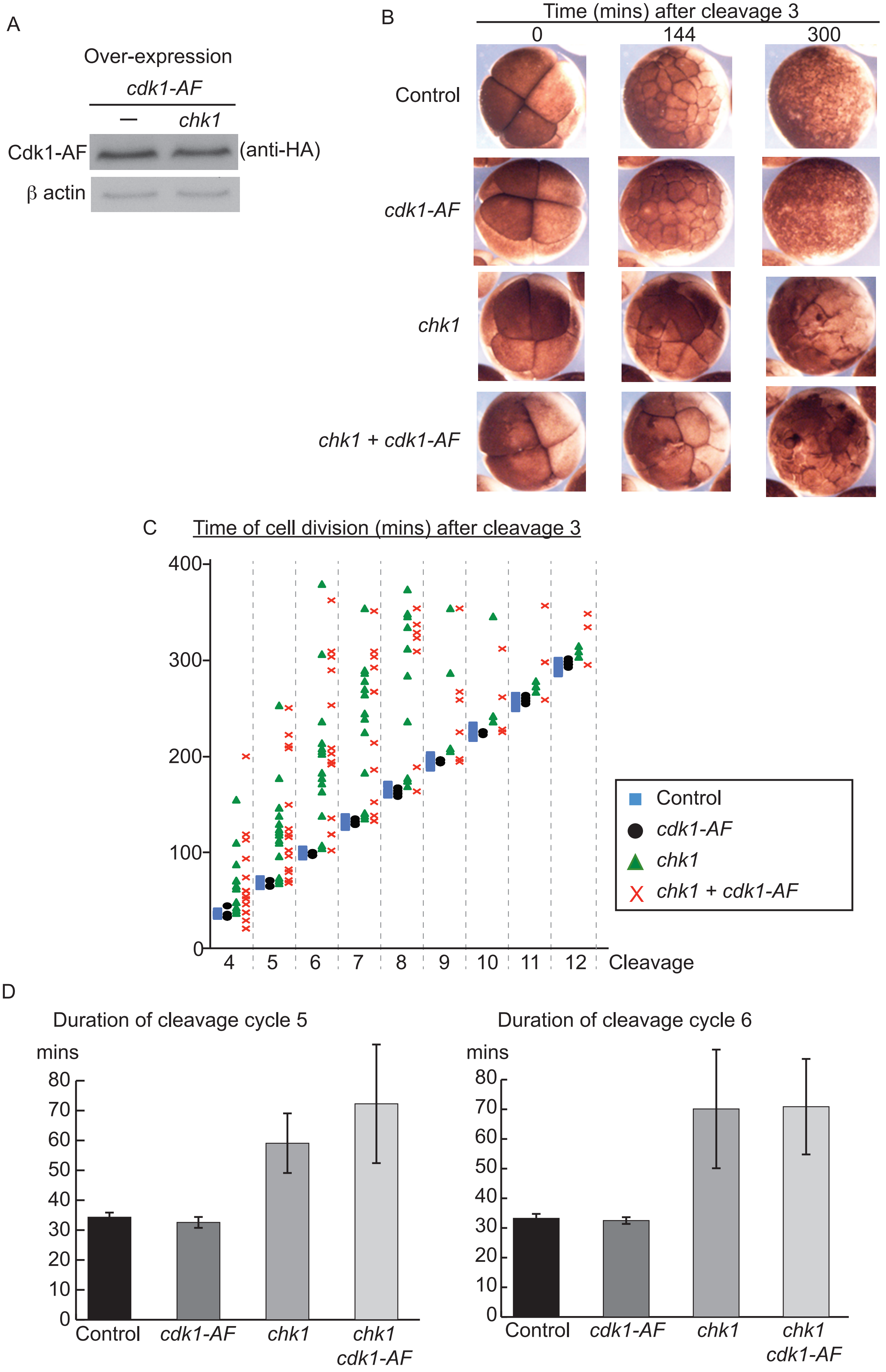

Figure S5

Over-expression of *cdk1-AF* does not affect the elongation of the cell cycle at the MBT

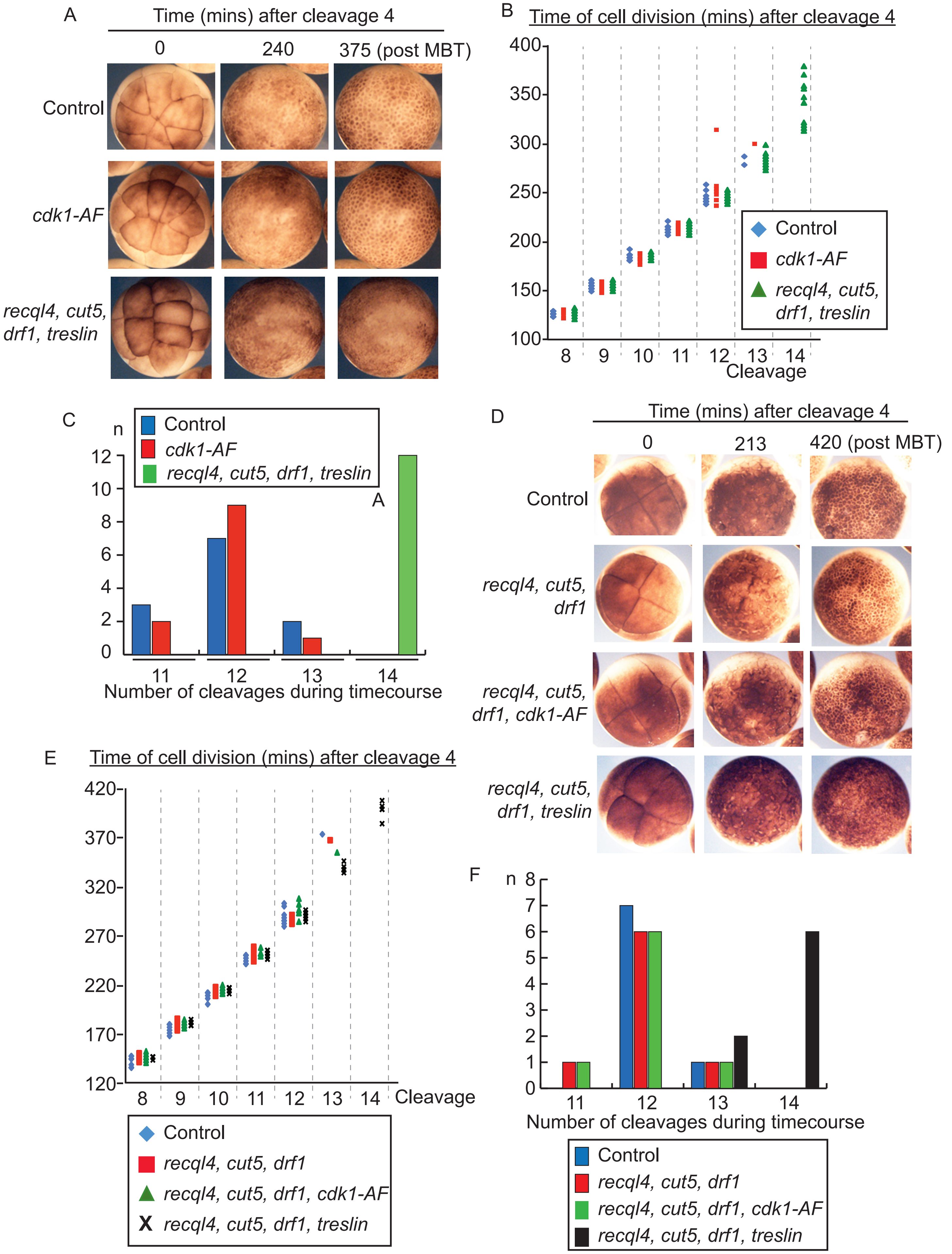

Figure S6
Over-expression of β *trcp* causes earlier Drf1 degradation and premature cell cycle elongation

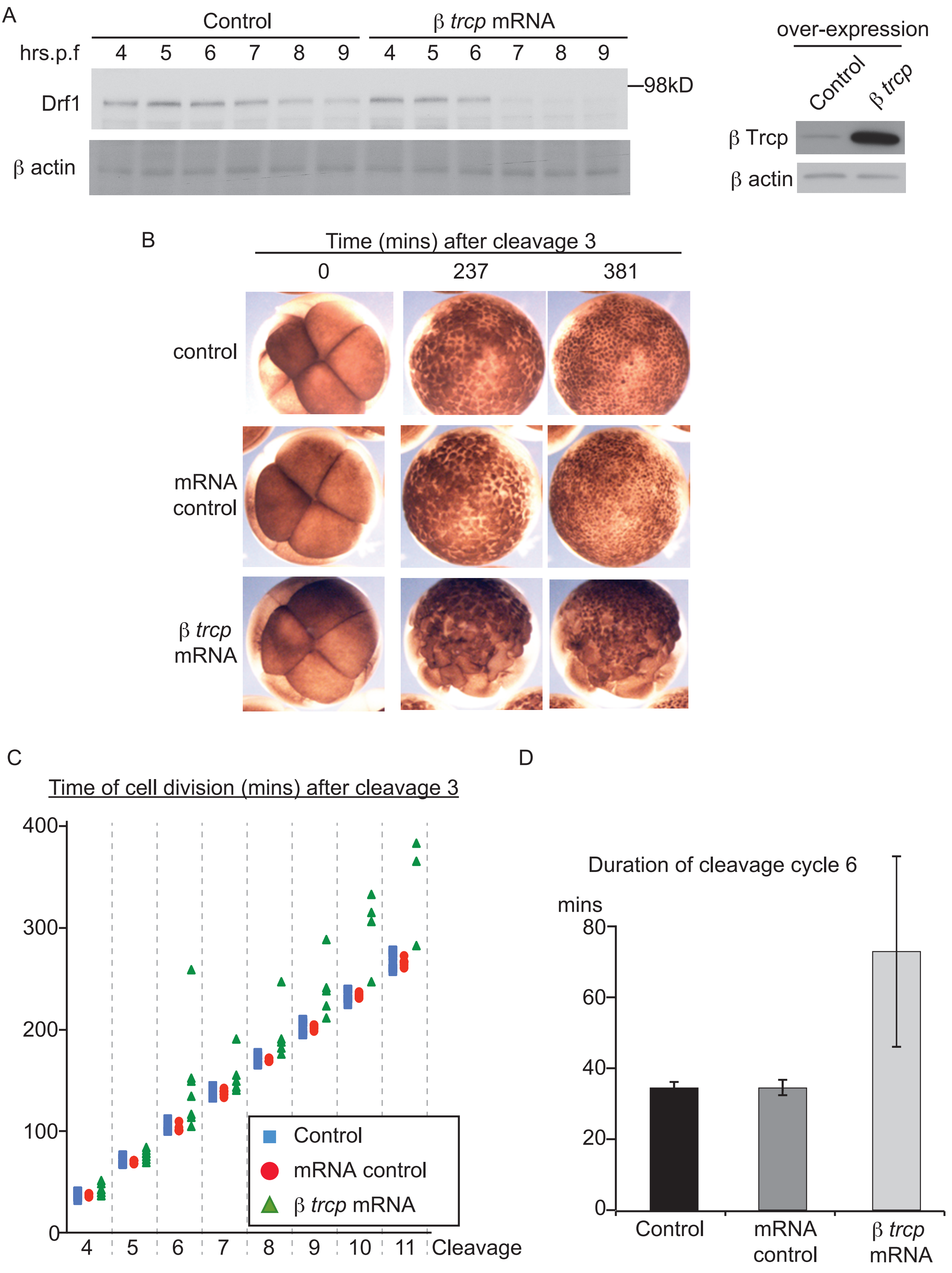

Figure S7
Unlike over-expression, stabilisation of Drf1 by down-regulation of β-*trcp* is not sufficient to allow rapid, synchronous divisions at the MBT.

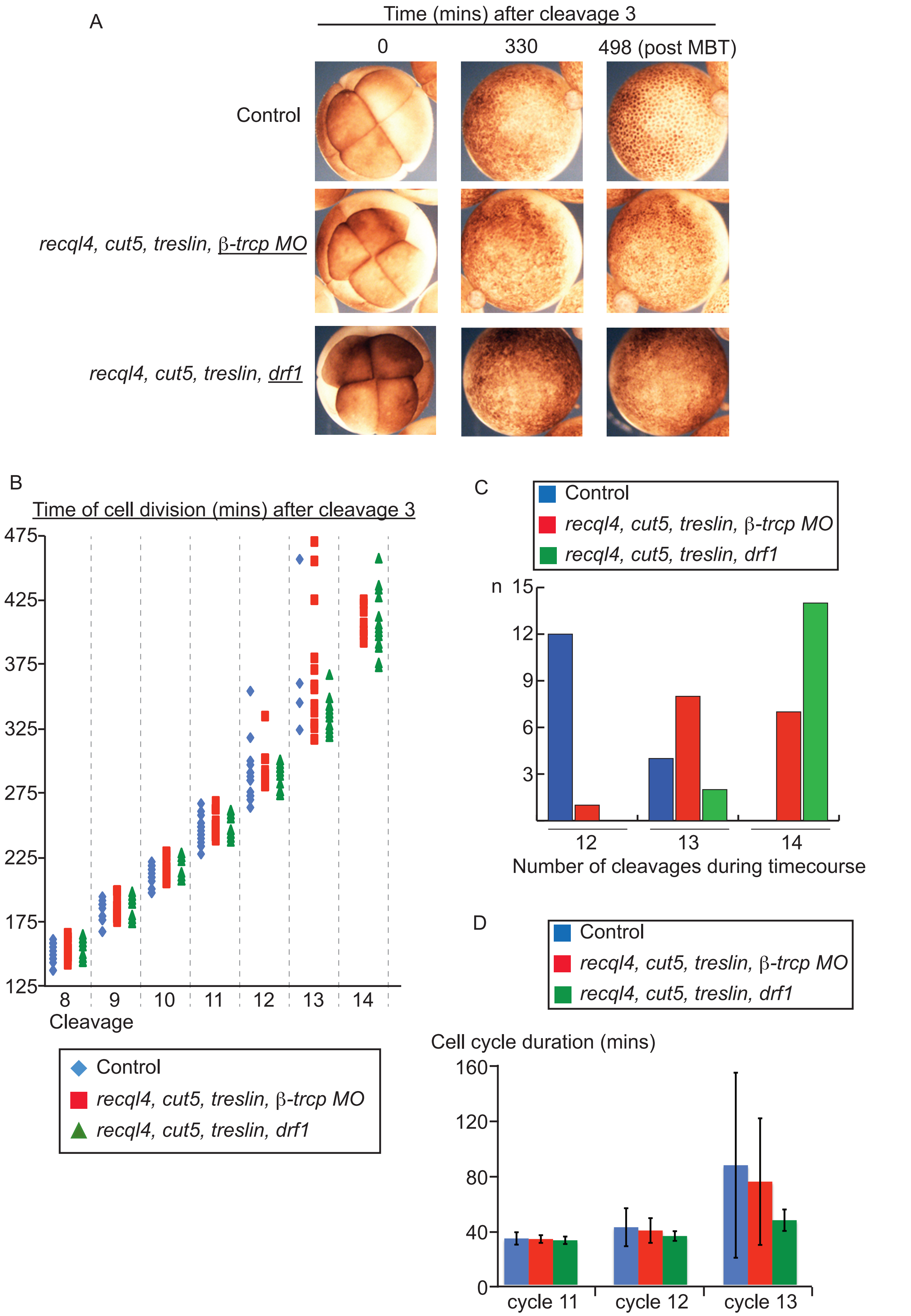

Supplementary figure legends

Figure S1. Chk1 inactivation blocks phosphoryation of downstream targets and Chk1 over-expression inhibits cell cycle progression in the early embryo. Related to Figure 1 and 3.

A. A Myc tagged fragment of Chk1 lacking the kinase domain (Chk1ΔKD) is phosphorylated by endogenous Chk1, resulting in a mobility shift in SDS-PAGE. This is an anti-myc Western blot of Chk1ΔKD from staged embryos at the indicated number of hours post fertilisation (hrs.p.f). Embryos were injected at the one cell stage either with water (control) or with mRNA corresponding to the *chk1* dominant negative mutant (D148A).

B. The DNA content of embryos 2hrs post MBT, injected at the one-cell stage with either water or *chk1 D148A* mRNA, was quantified on agarose gels (left) using ImageJ. The DNA content of 3 embryos was averaged and the control embryo DNA content set to 1 (right). Data are represented as mean ± SD

C. As in A except wild type *chk1* mRNA (200pg) was injected.

D. Images of embryos injected at the 1-cell stage either with water (control) or with increasing amounts of wild type *chk1* mRNA. Images are from time-lapse movies of these embryos at the indicated times after cleavage 3 (8 cell embryo).

E. As in A except embryos were incubated with 20mM HU with or without injection of mRNA for *drf1* or *dbf4.*

Figure S2. Over-expression of Treslin, Recq4 and Cut5 is required for rapid cell divisions at the MBT in embryos expressing Chk1 D148A. Related to Figure 2.

A. Embryos were injected in both blastomeres at the 2-cell stage with the indicated mRNA or water (control) and followed by time-lapse imaging. The 4th division, generating the 16-cell embryo was set to time zero. See also Movie S3.

B. The division of individual cells from A were followed throughout the movie. Each time-point represents the division of a single cell. Cleavages 4-7 are excluded for simplicity. n=24 cells from 6 embryos for each condition.

C. Total number of divisions each cell in B undergoes until the end of the time-lapse movie. The colour code is the same for both B and C.

Figure S3. Injection of aphidicolin is sufficient to block cell division before the MBT. Related to Figure 3.

A. Water (control), DMSO or aphidicolin was injected in both blastomeres at the 2-cell stage and followed by time-lapse imaging. The 3rd division, generating the 8-cell embryo was set to time zero.

B. The division of individual cells from A were followed throughout the movie. Each time-point represents the division of a single cell. n=12 cells from 3 embryos for each condition.

C,D as A,B.

E. Quantitation of the average duration of cell cycle 4 from C,D. n=12 cells from 3 embryos for each condition. Data are represented as mean ± SD, which indicates the synchrony of cell divisions.

Figure S4. Over-expression of Cdk1-AF does not prevent premature cell cycle lengthening after over-expression of Chk1. Related to Figure 3.

A. Anti HA western blot of N-terminally HA tagged Cdk1-AF from embryos 3 hrs.p.f.

B. Water (control), *chk1* mRNA, *cdk1-AF* mRNA or both mRNAs were injected in both blastomeres at the 2-cell stage and followed by time-lapse imaging. Total mRNA injected for *chk1* was 50pg, while for *cdk1-AF* it was 500pg. The 3rd division, generating the 8-cell embryo was set to time zero.

C. The division of individual cells from B were followed throughout the movies. Each time-point represents the division of a single cell. n=16 cells from 4 embryos for each condition.

D. Quantitation of the average duration of cell cycle 5 (left) and cycle 6 (right) from C. Data are represented as mean ± SD, which indicates the synchrony of cell divisions.

Figure S5. Over-expression of Cdk1-AF does not affect the duration of the cell cycle at the MBT. Related to Figure 3.

A. Embryos were injected in both blastomeres at the 2-cell stage with *cdk1-AF* mRNA (1ng total), the four limiting replication factors (300pg each total) or water (control) and followed by time-lapse imaging. The 4th division, generating the 16-cell embryo was set to time zero.

B. The division of individual cells from A were followed throughout the movies. Each time-point represents the division of a single cell. n=12 cells from 3 embryos for each condition.

C. Total number of divisions each cell in B undergoes until the end of the time-lapse movie. The colour code is the same for both B and C.

D. Embryos were injected in both blastomeres at the 2-cell stage with mRNAs or water (control) as indicated and followed by time-lapse imaging. The amounts of injected mRNAs were 500ng total for *cdk1-AF* and 300pg each total for *drf1, recq4*, *cut5* and *treslin*. The 4th division, generating the 16-cell embryo was set to time zero.

E. The division of individual cells from D were followed throughout the movies. Each time-point represents the division of a single cell. n=8 cells from 2 embryos for each condition.

F. Total number of divisions each cell in E undergoes until the end of the time-lapse movie. The colour code is the same for both E and F.

Figure S6. Over-expression of β-Trcp causes premature cell cycle elongation. Related to Figure 4.

A. (right) Anti β-Trcp western blot from stage 7 embryos (just before MBT) after injection at the 1 cell stage with water (control) or 1ng of *β-trcp* mRNA. (left) Anti-Drf1 western blot from staged embryos.

B. Water (control), antisense *β-trcp* mRNA (mRNA control) or *β-trcp* mRNA were injected in both blastomeres at the 2-cell stage and followed by time-lapse imaging. The total amount of injected mRNA was 5ng. The 3rd division, generating the 8-cell embryo was set to time zero.

C. The division of individual cells from B were followed throughout the movies. Each time-point represents the division of a single cell. n=8 cells from 2 embryos for each condition.

D. Quantitation of the average duration of cell cycle 6 from C. Data are represented as mean ± SD, which indicates the synchrony of cell divisions.

Figure S7. Over-expression of Drf1 is required to allow rapid, synchronous divisions at the MBT. Related to Figure 7D

A. Embryos were injected in both blastomeres at the 2-cell stage with mRNAs, anti-*β-trcp* morpholinos (MO) or water (control) as indicated and followed by time-lapse imaging. The total amounts of injected mRNAs/MO were *drf1, recq4*, *cut5* and *treslin* (300pg each), *β-trcp* morpholinos (80ng). The 3rd division, generating the 8-cell embryo was set to time zero.

B. The division of individual cells from A were followed throughout the movies. Each time-point represents the division of a single cell. n=16 cells from 4 embryos for each condition.

C. Total number of divisions each cell in B undergoes until the end of the time-lapse movie. The colour code is the same for B-D.

D. Quantitation of the average duration of cell cycles 11-13 from B. Data are represented as mean ± SD, which indicates the synchrony of cell divisions. Notably only over-expression of Drf1 (together with the other 3 limiting factors) allows for rapid and synchronous cleavages in cycles 12/13.
