# Supplementary figures and images for: Chk1 Inhibition of the Replication Factor Drf1 Guarantees Cell-Cycle Elongation at the Xenopus laevis Mid-blastula Transition
Source: Dev Cell. 2017 Jul 10;42(1):82–96.e3. doi: 10.1016/j.devcel.2017.06.010 (PMC5505860; doi:10.1016/j.devcel.2017.06.010)

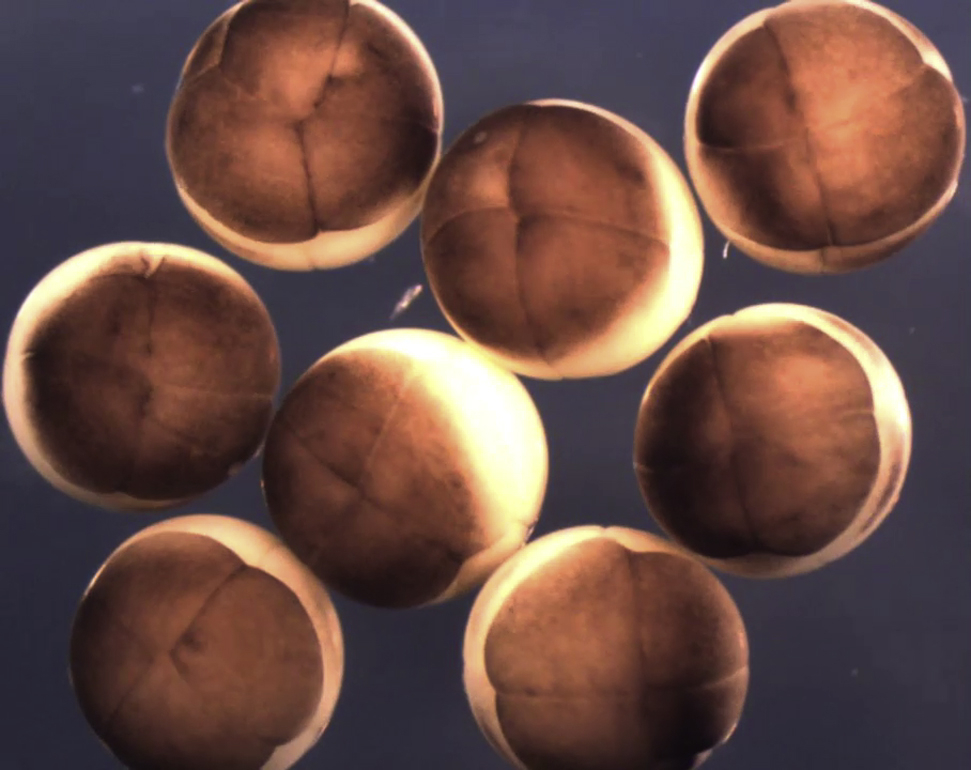

Supplement: Movie S1. Chk1 Inhibition Does Not Affect the Cell Cycles at the MBT, Related to Figure 1 — The top 4 embryos were control (water) injected, the 2 embryos below that were injected with chk1 D148A mRNA and the bottom 2 embryos were injected with mRNA for cut5, drf1, treslin and recq4. [file mmc2.jpg]

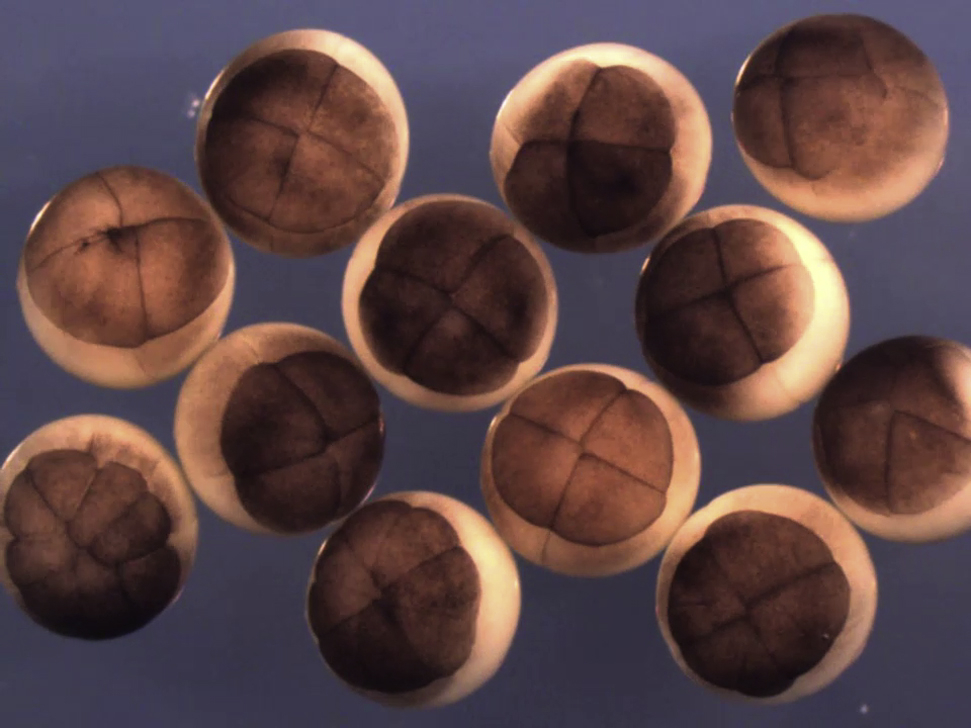

Supplement: Movie S2. Chk1 Inhibits Drf1 at the MBT, Related to Figure 2 — The top 3 embryos were control (water) injected, the 3 embryos below that were injected with chk1 D148A mRNA, the 3 embryos below that were injected with mRNA for cut5, treslin and recq4 and the bottom 3 embryos were injected with mRNA for cut5, treslin and recq4 and chk1 D148A. [file mmc3.jpg]

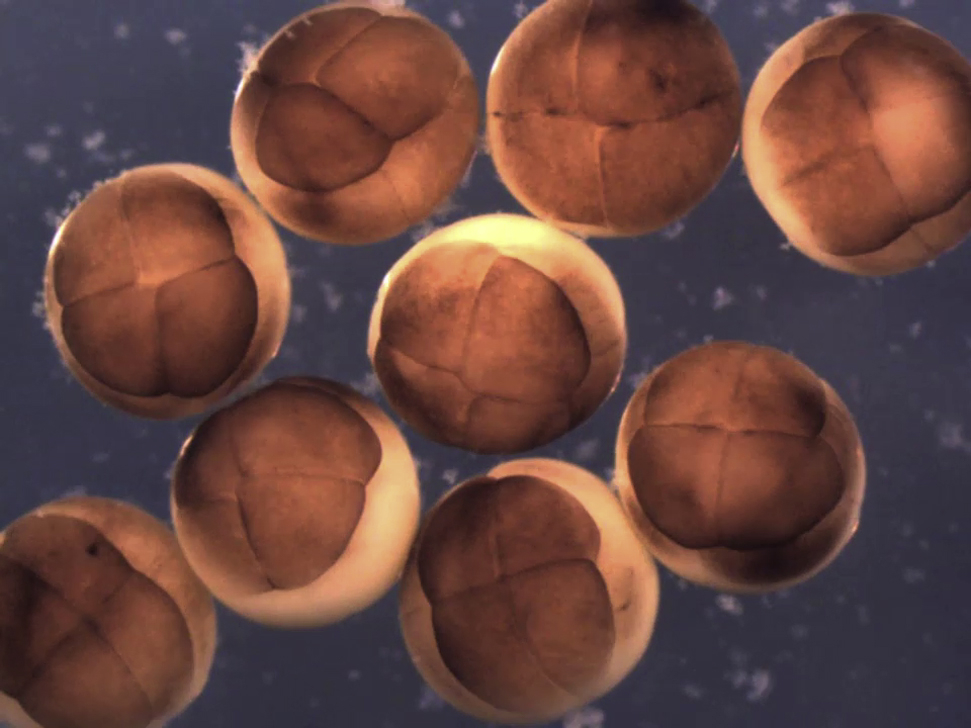

Supplement: Movie S3. Over-expression of Treslin, Recq4, and Cut5 Is Required for Rapid Cell Divisions at the MBT in Embryos Expressing Chk1 D148A, Related to Figure S2 — The embryo in the top right was control (water) injected, the 2 embryos top/centre were injected with mRNA for chk1 D148A, treslin and recq4, the 2 embryos below that were injected with mRNA for chk1 D148A, treslin and cut5, the 2 embryos below that were injected with mRNA for chk1 D148A, recq4 and cut5 and the bottom 2 embryos were injected with mRNA for cut5, treslin and recq4 and chk1 D148A. [file mmc4.jpg]

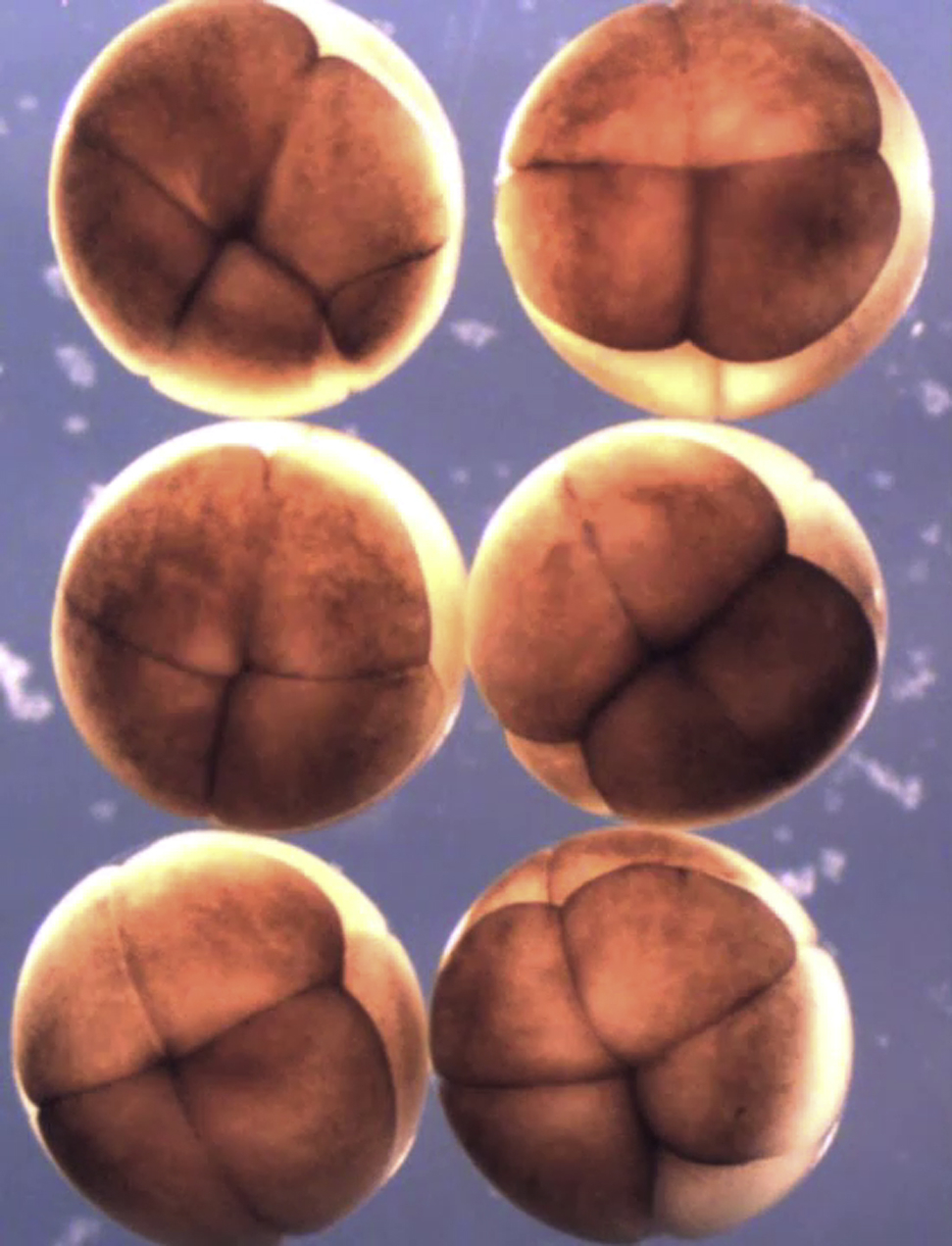

Supplement: Movie S4. β-TRCP Controls Drf1 Levels, Related to Figure 4 — The top 2 were injected with control morpholino, the 2 central embryos were injected with the anti β-trcp morpholino and the bottom 2 embryos were injected with the anti β-trcp morpholino plus mRNA for cut5, treslin and recq4. [file mmc5.jpg]

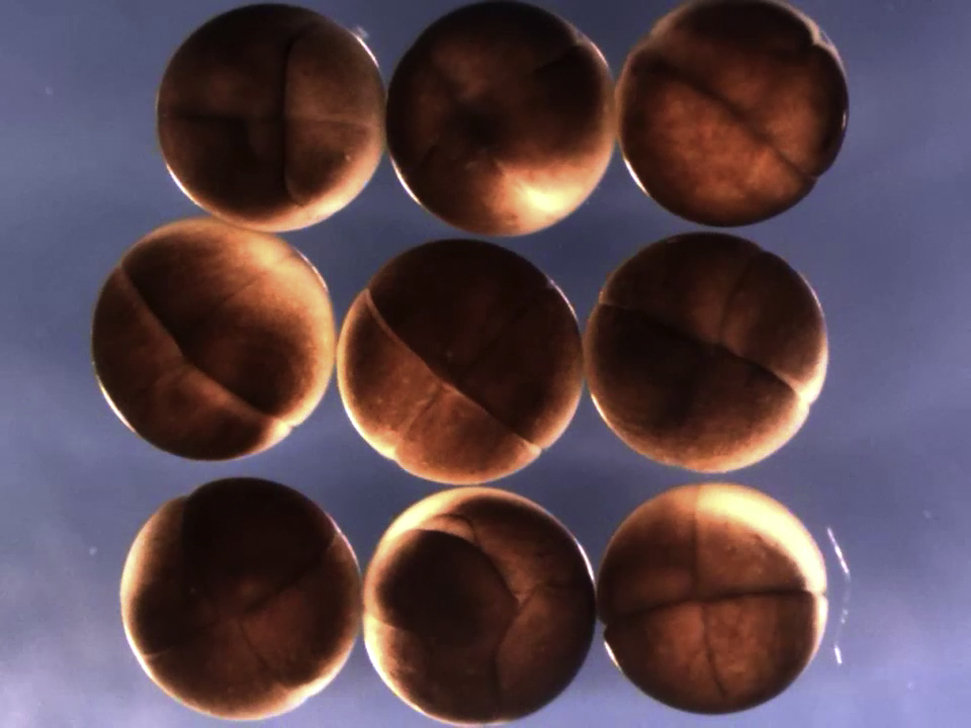

Supplement: Movie S5. Chk1 Blocks the Cell Cycle by Inhibiting DDK, Related to Figure 3 — The top 3 embryos were injected with chk1 mRNA(50 pg), the middle 3 with chk1 (50 pg) + drf1 (500 pg) and the bottom 3 with chk1 (50 pg) + dbf4 (500 pg). [file mmc6.jpg]
