# Supplementary material for: Chk1 Inhibition of the Replication Factor Drf1 Guarantees Cell-Cycle Elongation at the Xenopus laevis Mid-blastula Transition
Source: Dev Cell. 2017 Jul 10;42(1):82–96.e3. doi: 10.1016/j.devcel.2017.06.010 (PMC5505860; doi:10.1016/j.devcel.2017.06.010)
Supplement: Document S2. Article plus Supplemental Information [file mmc7.pdf]

Article

Developmental Cell

Chk1 Inhibition of the Replication Factor Drf1 Guarantees Cell-Cycle Elongation at the *Xenopus laevis* Mid-blastula Transition

Graphical Abstract

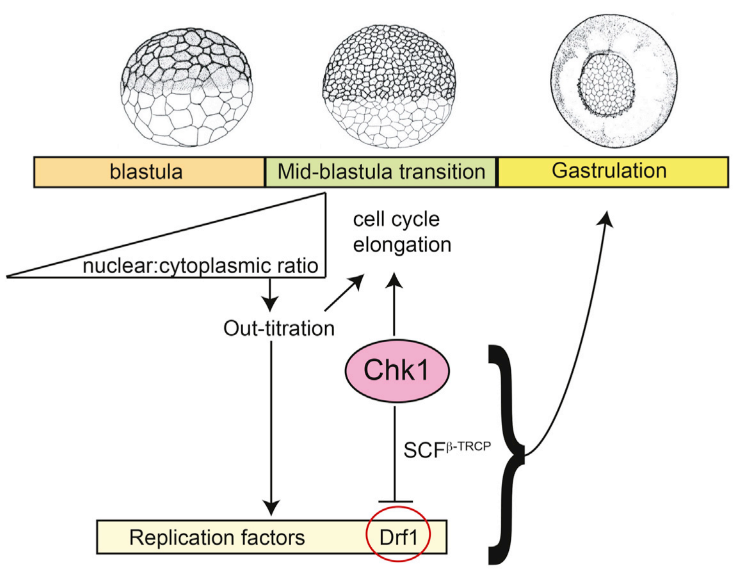

Authors

Clara Collart, James C. Smith, Philip Zegerman

Correspondence

p.zegerman@gurdon.cam.ac.uk

In Brief

Embryonic divisions lengthen at the mid-blastula transition (MBT) in many metazoa. Collart et al. show in *Xenopus laevis* that the checkpoint kinase Chk1 causes SCF$^{\beta\text{-TRCP}}$-dependent degradation of the replication factor Drf1 at the MBT. Inhibition of Drf1 is an essential function of Chk1, guaranteeing timely cell-cycle elongation.

Highlights

- Activation of Chk1 at the *Xenopus* MBT results in the degradation of Drf1
- Drf1 degradation is SCF$^{\beta\text{-TRCP}}$ dependent
- Chk1 blocks the cell cycle in the early embryo through inhibition of Drf1
- Inhibition of Drf1 is an essential function of Chk1 during gastrulation

Collart et al., 2017, Developmental Cell *42*, 82–96
July 10, 2017 © 2017 The Authors. Published by Elsevier Inc.
http://dx.doi.org/10.1016/j.devcel.2017.06.010

Developmental Cell
Article

Chk1 Inhibition of the Replication Factor Drf1 Guarantees Cell-Cycle Elongation at the *Xenopus laevis* Mid-blastula Transition

Clara Collart,[1,2] James C. Smith,[2] and Philip Zegerman[1,3,*]
[1]Department of Biochemistry, Wellcome Trust/Cancer Research UK Gurdon Institute, The Henry Wellcome Building of Cancer and Developmental Biology, University of Cambridge, Cambridge CB2 1QN, UK
[2]Developmental Biology Laboratory, Francis Crick Institute, Midland Road, London NW1 1AT, UK
[3]Lead Contact
*Correspondence: p.zegerman@gurdon.cam.ac.uk
http://dx.doi.org/10.1016/j.devcel.2017.06.010

SUMMARY

The early cell divisions of many metazoan embryos are rapid and occur in the near absence of transcription. At the mid-blastula transition (MBT), the cell cycle elongates and several processes become established including the onset of bulk transcription and cell-cycle checkpoints. How these events are timed and coordinated is poorly understood. Here we show in *Xenopus laevis* that developmental activation of the checkpoint kinase Chk1 at the MBT results in the SCF$^{\beta\text{-TRCP}}$-dependent degradation of a limiting replication initiation factor Drf1. Inhibition of Drf1 is the primary mechanism by which Chk1 blocks cell-cycle progression in the early embryo and is an essential function of Chk1 at the blastula-to-gastrula stage of development. This study defines the downregulation of Drf1 as an important mechanism to coordinate the lengthening of the cell cycle and subsequent developmental processes.

INTRODUCTION

The early embryonic development of many animals, particularly those that develop externally, involves a rapid expansion in cell numbers. These fast early cell divisions exhibit very little zygotic transcription and rely on maternally supplied products (Langley et al., 2014; Tadros and Lipshitz, 2009). After a species-specific number of rapid divisions, the cell cycle elongates and the zygotic transcriptional program is established. This developmental event is called the mid-blastula transition (MBT) or the maternal to zygotic transition (MZT; Tadros and Lipshitz, 2009). The MBT is also the point when many additional cellular processes are coordinated, including the onset of cell-cycle checkpoints, apoptosis, and cell motility (Farrell and O'Farrell, 2014; Hensey and Gautier, 1997; Kane and Kimmel, 1993; Newport and Kirschner, 1982a, 1982b). How the events of the MBT are timed and coordinated is poorly understood, yet these processes are critical for subsequent gastrulation when the three germ layers of the embryo are formed.

One mechanism responsible for timing the events of the MBT involves the nuclear to cytoplasmic (N/C) ratio (Ferree et al., 2016; Kane and Kimmel, 1993; Newport and Kirschner, 1982a). In the virtual absence of growth and zygotic transcription, the early cell-cleavage divisions result in an exponential increase in the ratio of DNA to cytoplasm. The N/C ratio has been proposed to act as a timing mechanism by switching on cellular responses when particular maternally deposited components become critically limiting. Such an N/C ratio timer has been shown in frog, fish, and fly embryos to be responsible for coordinating cell-cycle changes, checkpoint activation, and the transcription of subsets of zygotic genes (Ferree et al., 2016; Gotoh et al., 2011; Kane and Kimmel, 1993; Newport and Kirschner, 1982a). The N/C ratio is not the only timer required to trigger the MBT, as several events occur independently of DNA content including the degradation of maternal mRNA (Tadros and Lipshitz, 2009) and the downregulation of cyclin E in *Xenopus* (Howe and Newport, 1996).

The lengthening of the cell cycle at the MBT in flies and frogs coincides with changes in DNA replication dynamics and decreased rates of replication initiation (Hyrien et al., 1995; Shermoen et al., 2010). We have shown in *Xenopus laevis* embryos that the increasing N/C ratio titrates out four replication initiation factors Drf1, Treslin, Recq4, and Cut5 (Collart et al., 2013). Over-expression of these factors in *Xenopus* embryos sustains high rates of replication initiation, which is sufficient to allow the continuation of rapid cleavage divisions after the MBT at least during cycles 12–15 (Collart et al., 2013). Importantly the number of rapid cleavage divisions induced by these factors after the MBT is closely linked to their protein levels, supporting the idea that titration of these chromatin binding factors by the increasing N/C ratio acts as a timer governing cell-cycle duration (Collart et al., 2013). For the cell cycle to lengthen after precisely the correct number of cycles, the amounts of these four factors must therefore be strictly controlled during early embryogenesis, but how this is achieved is not clear.

One of the events of the MBT is the activation of the checkpoint kinase Chk1 (Shimuta et al., 2002; Sibon et al., 1997), which is essential for early embryogenesis across metazoa (Fogarty et al., 1994; Kalogeropoulos et al., 2004; Liu et al., 2000; Shimuta et al., 2002; Takai et al., 2000). Interestingly, over-expression of Drf1, Treslin, Recq4, and Cut5, which causes rapid cell cycles at the MBT, also leads to increased and premature Chk1 activation, due in part to depletion of deoxynucleotide triphosphate pools (Collart et al., 2013). In other systems such as in mammalian cells, Chk1 is known to inhibit cell-cycle progression either by blocking entry into mitosis through regulation of CDK activity

 © 2017 The Authors. Published by Elsevier Inc.
This is an open access article under the CC BY license (http://creativecommons.org/licenses/by/4.0/).

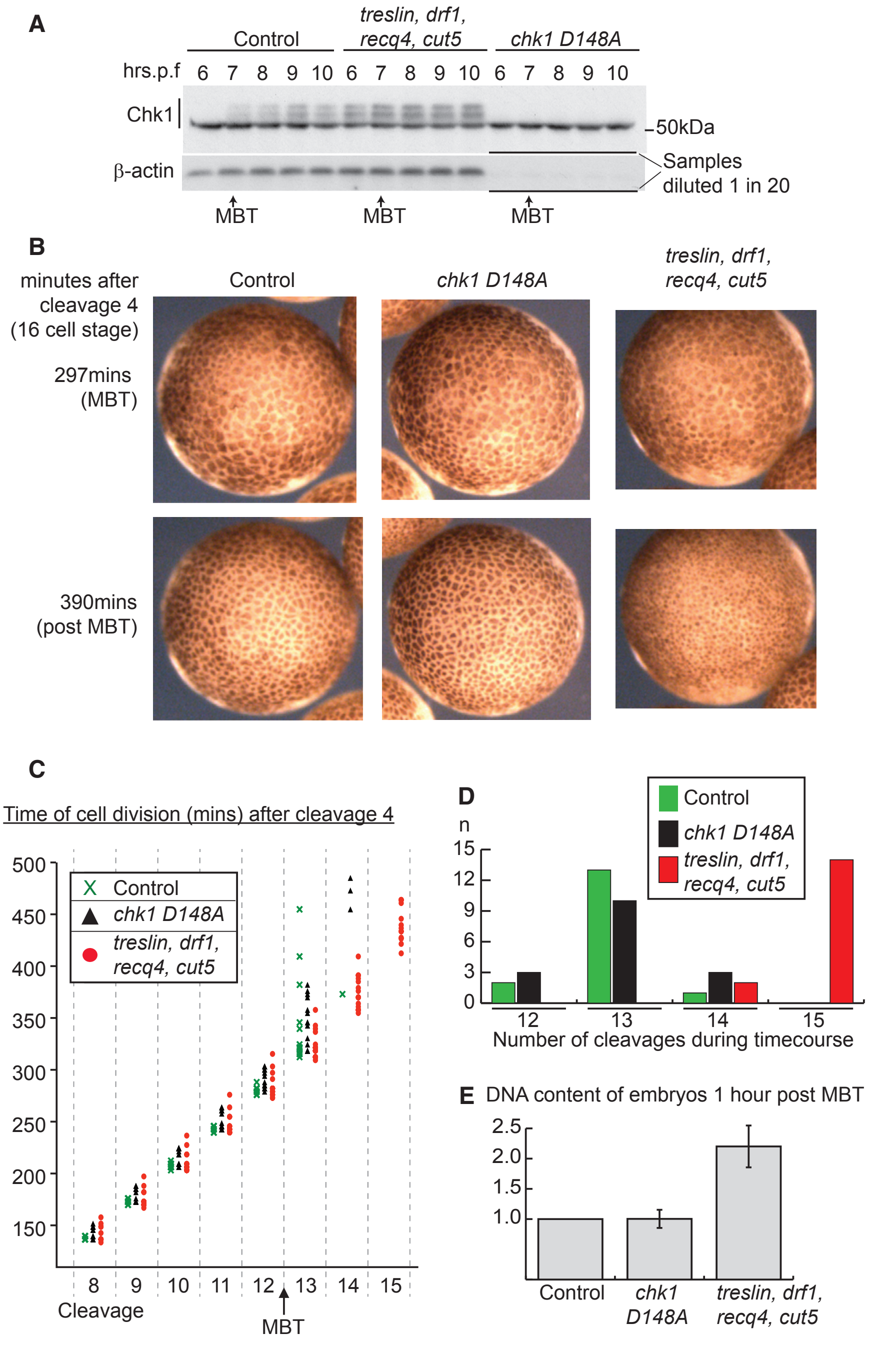

Figure 1. Chk1 Inhibition Does Not Affect the Cell Cycles at the MBT

(A) Western blot of Chk1 and β-actin from staged embryos at the indicated number of hours post fertilization (hrs.p.f). Embryos were injected at the one cell stage either with water (control) or with mRNA of the four limiting replication factors (*treslin*, *drf1*, *recq4*, and *cut5*) or the *chk1* dominant-negative mutant (D148A). The extracts from embryos over-expressing Chk1 D148A were diluted 1 in 20 to allow a direct comparison between endogenous and over-expressed Chk1. See also Figure S1A.

(B) Still images from time-lapse movies of embryos injected in both blastomeres at the 2-cell stage as in (A) The fourth division, generating the 16-cell embryo, was set to time zero. See also Movie S1.

(C) The division of embryonic cells from (B) were followed throughout the movie. Each time point represents the division of a single cell. The cell divisions for the three conditions are displayed side by side for each cleavage cycle. Cleavages 4–7 are excluded for simplicity. n = 16 cells from four embryos for each condition.

(D) Total number of divisions undergone by each cell in (C) until the end of the time-lapse movie.

(E) The DNA content of embryos, injected as in (A), was quantified on agarose gels using ImageJ. The DNA content of control embryos was set to 1. Data are presented as mean ± SD, n = 5. See also Figure S1B.

(Bartek et al., 2004) or by inhibiting DNA replication (Maya-Mendoza et al., 2007). We therefore set out to understand how *X. laevis* embryos over-expressing limiting replication factors have fast cell cycles at the MBT even though Chk1 is active.

Our results show that Chk1 regulates the abundance of the replication factor Drf1 at the MBT through phospho-dependent degradation by the SCF$^{\beta\text{-TRCP}}$ E3 ubiquitin ligase. Through manipulation of both Chk1 and SCF$^{\beta\text{-TRCP}}$ activities we demonstrate that this pathway guarantees the lengthening of the cell cycle by ensuring that Drf1 levels become critically limiting at the correct stage of development. Inhibition of Drf1 is the primary mechanism by which Chk1 inhibits the cell cycle in the early embryo, and we show that this is an essential function for Chk1 during blastula-to-gastrula stages. Together, the results of this study uncover a mechanism to ensure that the egg is subdivided into the exact number of cells during normal embryogenesis and provide insight into how events at the MBT are coordinated.

RESULTS

Chk1 Inhibition Alone Does Not Affect the Cell Cycle at the MBT

In normal *X. laevis* embryos developing at 20°C, the MBT occurs at 6.5–7.5 hr post fertilization. This event is marked by the transient developmental phosphorylation and activation of the checkpoint kinase Chk1 (Shimuta et al., 2002; Figure 1A). We have previously shown that over-expression of the limiting replication initiation factors Drf1, Treslin, Recq4, and Cut5, which causes continuation of rapid, synchronous cleavage divisions during the MBT, leads to earlier and increased Chk1 activation (Figure 1A; Collart et al., 2013). Since embryos over-expressing limiting replication factors have fast cell cycles at the MBT despite earlier Chk1 activation, we wondered what role Chk1 might play in controlling the embryonic cell cycle.

To test the role of Chk1 in the early embryonic divisions in *X. laevis*, we over-expressed a dominant-negative, kinase-dead mutant of *chk1* (D148A, Shimuta et al., 2002) by injection of mRNA into 1-cell embryos. Over-expression of this mutant abrogates the phosphorylation of Chk1 targets including Cdc25 (Uto et al., 2004) and itself (Figure S1A) and as a result this Chk1 D148A mutant did not exhibit an activation-dependent mobility shift at the MBT (Figure 1A). To assess the effects of Chk1 inhibition on the cell cycle, we analyzed movies of embryos over-expressing this kinase-dead mutant (Movie S1 and Figure 1B). To quantify these movies and standardize our analyses between embryos, we set the fourth cleavage (16-cell embryo) to time zero and followed the timing of division of individual blastomeres (Figure 1C). In addition, we measured the total number of cleavages undergone by each blastomere throughout the length of the movie (Figure 1D). Together, these data provide the timing and frequency of division of cells in live embryos.

While the cell cycles in control embryos slowed at the MBT after cleavage 12 (green crosses, Figure 1C), over-expression of limiting replication factors caused an increased number of synchronous cleavages at the MBT (Figures 1C and 1D), as expected (Collart et al., 2013). As a result, post-MBT embryos that over-express these limiting factors have more cells, which are smaller in size than in control embryos (Figure 1B). By contrast, over-expression of the kinase-dead *chk1* had little effect on cell-cycle duration or the total number of divisions, and these embryos resembled controls after the MBT (Figures 1B–1D). A previous study using the same *chk1 D148A* allele inferred that Chk1 inhibition was sufficient to allow extra cell divisions after the MBT through measurement of the DNA content of the embryo (Shimuta et al., 2002). To explore this discrepancy, we analyzed the DNA content of embryos post MBT (Figures 1E and S1B). Consistent with our cytological analyses, embryos over-expressing limiting replication initiation factors, which have approximately doubled their cell numbers relative to controls 1 hr post MBT (Figure 1C), had also doubled their DNA content, whereas Chk1 D184A over-expressing embryos had not (Figures 1E and S1B). From this we conclude that Chk1 inhibition alone has little effect on the cell cycle at the MBT in *X. laevis*. We are not sure why a previous study (Shimuta et al., 2002) reached a different conclusion using the same *chk1* allele at similar levels of over-expression.

Since Chk1 inhibition does not affect the elongation of the cell cycle in *Xenopus*, at least during cycles 12–15 (Figure 1), we wondered whether Chk1 activity regulates the cell cycle at all in this organism. In line with previous studies (Kappas et al., 2000), over-expressed wild-type *chk1* was active in the early embryo and robustly inhibited cell-cycle progression (Figures S1C and S1D). We therefore set out to reconcile how embryos over-expressing limiting replication factors have fast cell cycles even though Chk1 has even higher than normal levels of activation (Figure 1A).

Chk1 Inhibits the Limiting Replication Factor Drf1 at the MBT

We have previously shown that Rad53, the functionally analogous kinase to Chk1 in budding yeast, blocks S-phase progression by inhibiting two replication initiation factors, Dbf4 and Sld3 (Zegerman and Diffley, 2010). *Xenopus* Treslin is orthologous to yeast Sld3 (Kumagai et al., 2011) and there are two *Xenopus* orthologs of Dbf4 (Dbf4 and Drf1), with Drf1 being predominant during cleavage divisions (Silva et al., 2006; Takahashi and Walter, 2005). Since both Drf1 and Treslin are limiting replication initiation factors at the MBT in *X. laevis* (Collart et al., 2013), we wondered whether the normal function of Chk1 is to inhibit one or more of these limiting factors and that by over-expressing them we effectively bypass Chk1 function.

To test whether *Xenopus* Chk1 regulates Drf1, Dbf4 or Treslin, we analyzed the phosphorylation of these proteins at the MBT in the presence or absence of active Chk1. While we did not detect any Chk1-dependent modifications of Treslin or Dbf4 (Figure 2A and data not shown), we did observe that Drf1 abundance decreased dramatically after the MBT in *Xenopus* as previously described (Silva et al., 2006; Takahashi and Walter, 2005). Significantly, this downregulation of Drf1 was coincident with Chk1 activation and was dependent on Chk1 activity (Figure 2A).

If a role for Chk1 at the MBT is to limit Drf1 abundance, then we hypothesized that inhibition of Chk1 together with over-expression of the other three limiting factors, Cut5, Treslin, and Recq4, should permit the continuation of fast cell cycles at the MBT. As we have shown previously (Collart et al., 2013), over-expression of Cut5, Treslin, and Recq4 without Drf1 is not sufficient to drive fast cleavage divisions beyond the MBT, and these embryos resembled controls (Movie S2 and Figures 2B–2D). Importantly, however, when we combined over-expression of Chk1 D148A with over-expression of Cut5, Treslin, and Recq4, embryos underwent at least one extra division after the MBT, resulting in embryos with a greater number of smaller cells (Figures 2B–2D). From this we conclude that Chk1 is an inhibitor of Drf1 and that in the absence of Chk1 activation, Drf1 levels are sufficient for rapid S-phase progression at least during cycle 13 if the other three limiting replication factors are abundant.

Although our analyses in Figure 2A did not identify other potential targets of Chk1, it is feasible that developmental Chk1 activation leads to the inhibition of other replication factors, such as Treslin. If this were the case then we would expect that Chk1 inhibition would prevent such a factor becoming limiting at the MBT, as observed with Drf1 (Figures 2B–2D). To test this we over-expressed all combinations of just two of the limiting factors in embryos expressing Chk1 D148A. As shown in Movie S3 and Figure S2, only when Treslin, Recq4, and Cut5 were all over-expressed together with Chk1 D148A did extra divisions continue beyond the MBT. From this we conclude that Chk1 inhibits Drf1, but not the other three limiting factors at the MBT.

Chk1 Blocks Cell-Cycle Progression by Inhibition of DDK

Drf1 and Dbf4 bind to and activate Cdc7, to form the DDK (Dbf4-dependent kinase) complex, which is required for replication initiation (Labib, 2010). In pre-MBT embryos Drf1-Cdc7 is the predominant form of DDK, while after the MBT Drf1 is replaced by Dbf4 (Silva et al., 2006; Takahashi and Walter, 2005). Both Drf1-Cdc7 and Dbf4-Cdc7 facilitate the essential role of DDK in replication initiation (Silva et al., 2006; Takahashi and Walter, 2005) and either Drf1 or Dbf4 over-expression, together with

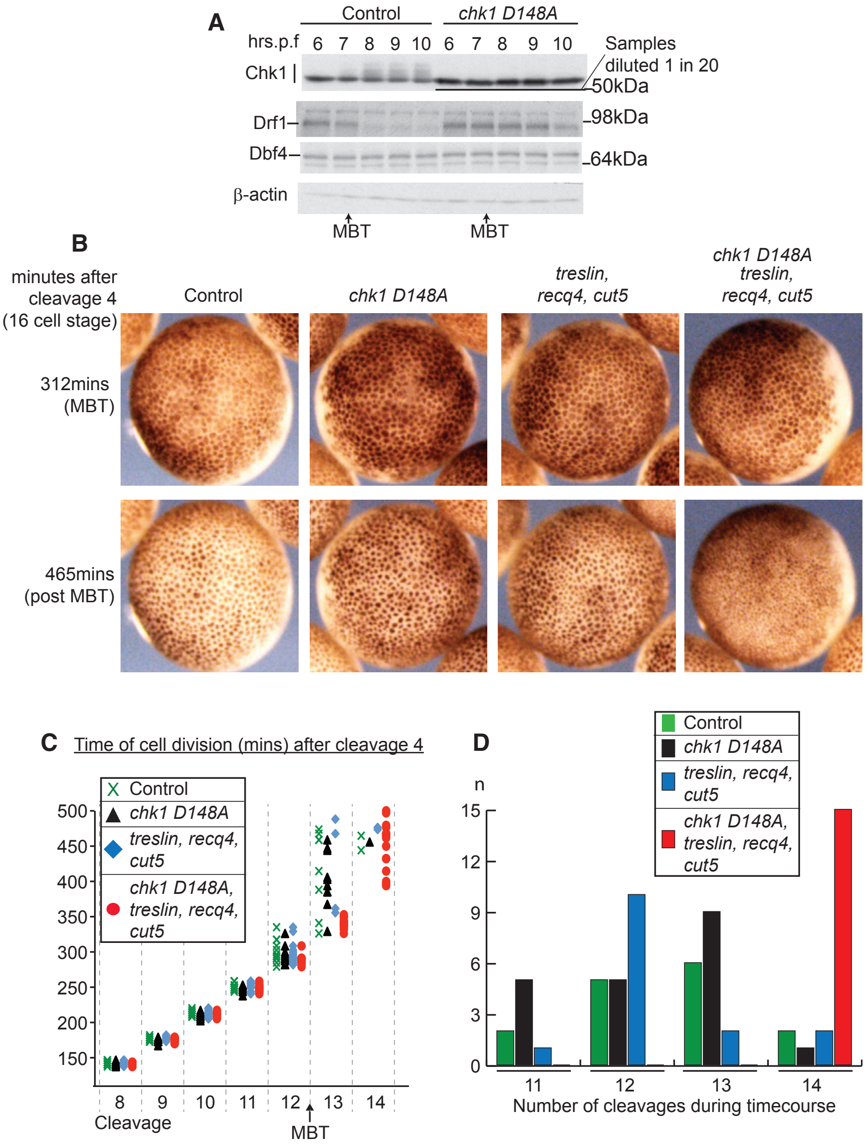

Figure 2. Chk1 Inhibits Drf1 at the MBT

(A) Western blot as in Figure 1A. For only the Chk1 blot from the over-expression of *chk1 D148A*, extracts were diluted 1 in 20 to allow a direct comparison between endogenous and over-expressed Chk1. All other samples are undiluted.

(B–D) As for Figures 1B–1D. For (C) and (D), n = 15 cells from four embryos for each condition.

See also Movies S2 and S3; Figure S2.

the other three limiting replication factors, can support fast cell cycles during the MBT (data not shown). These paralogs are therefore equivalent for their essential roles in replication initiation, yet are differently regulated by Chk1 (Figure 2A).

As Chk1 regulates Drf1 levels (Figure 2A) and premature Chk1 activation inhibits the cell cycle in pre-MBT embryos (Figure S1D; Kappas et al., 2000), we wondered whether this Chk1-mediated cell-cycle control might occur through inhibition of Drf1. To address this question we over-expressed wild-type *chk1* together with either *dbf4* or *drf1*. Over-expression of wild-type *chk1* resulted in cell-cycle arrest as expected (Figure 3A), but importantly this arrest was rescued by co-over-expression of Dbf4 and partially rescued by over-expression of Drf1 (Figure 3A).

To analyze this in more detail, we timed individual cleavage divisions in embryos. Chk1 over-expression greatly increased the length and asynchrony of cell divisions in the early embryo (Figure 3B [blue diamonds] and Movie S5). Both Drf1 and Dbf4 over-expression rescued the Chk1-dependent block to cell-cycle progression, resulting in shorter cycles with greater synchrony (Figure 3B). As in Figure 3A, Dbf4 was a better suppressor of Chk1-mediated cell-cycle arrest than Drf1 (Figure 3B). We presume that the difference in effectiveness between Dbf4 and Drf1 in rescuing the Chk1-mediated cell-cycle defect is due to the fact that Drf1 is inhibited by Chk1 while Dbf4 is not (Figure 2A and Discussion). By quantifying a single cleavage cycle (cycle 5) across multiple embryos, it is clear that Dbf4 over-expression is sufficient to return the cell-cycle duration (Figure 3C, red dotted line) and the synchrony of division (Figure 3C, error bars) back to wild-type levels after Chk1 over-expression. This rescue of ectopic Chk1 activation by Drf1 or Dbf4 is not because DDK is acting as an inhibitor of Chk1, as over-expression of Drf1 or Dbf4 does not inhibit Chk1 activity in *Xenopus* embryos (Figures 1A and S1E). The ability of Drf1 over-expression to bypass the Chk1-mediated cell-cycle inhibition likely explains how over-expression of the four limiting factors (including Drf1) is sufficient to allow fast cell cycles at the MBT regardless of Chk1 activation levels (Figure 1).

Drf1 and Dbf4 have equivalent roles in DNA replication as part of DDK, and both can rescue the cell-cycle arrest caused by Chk1 over-expression (Figure 3). These data therefore suggest that Chk1 blocks the cell cycle by inhibiting replication initiation through inhibition of DDK. Indeed, *Xenopus* Chk1 has been shown to be an inhibitor of DNA replication *in vitro* (Platel et al., 2015). Despite this, previous studies involving soaking embryos in replication inhibitors suggested that inhibition of DNA replication could not prevent cell-cycle progression before the MBT in *Xenopus* (Newport and Dasso, 1989). However, as shown in Figure S3, injection of the replication inhibitor aphidicolin into *Xenopus* embryos resulted in a robust block to cell-cycle progression. We therefore conclude that inhibition of DNA replication is sufficient to block cell-cycle progression in early *Xenopus* embryos. We suggest that the earlier studies (Newport and Dasso, 1989) may be misleading because the tight cell-cell junctions in pre-MBT embryos prevent the effective uptake of inhibitors from the surrounding media.

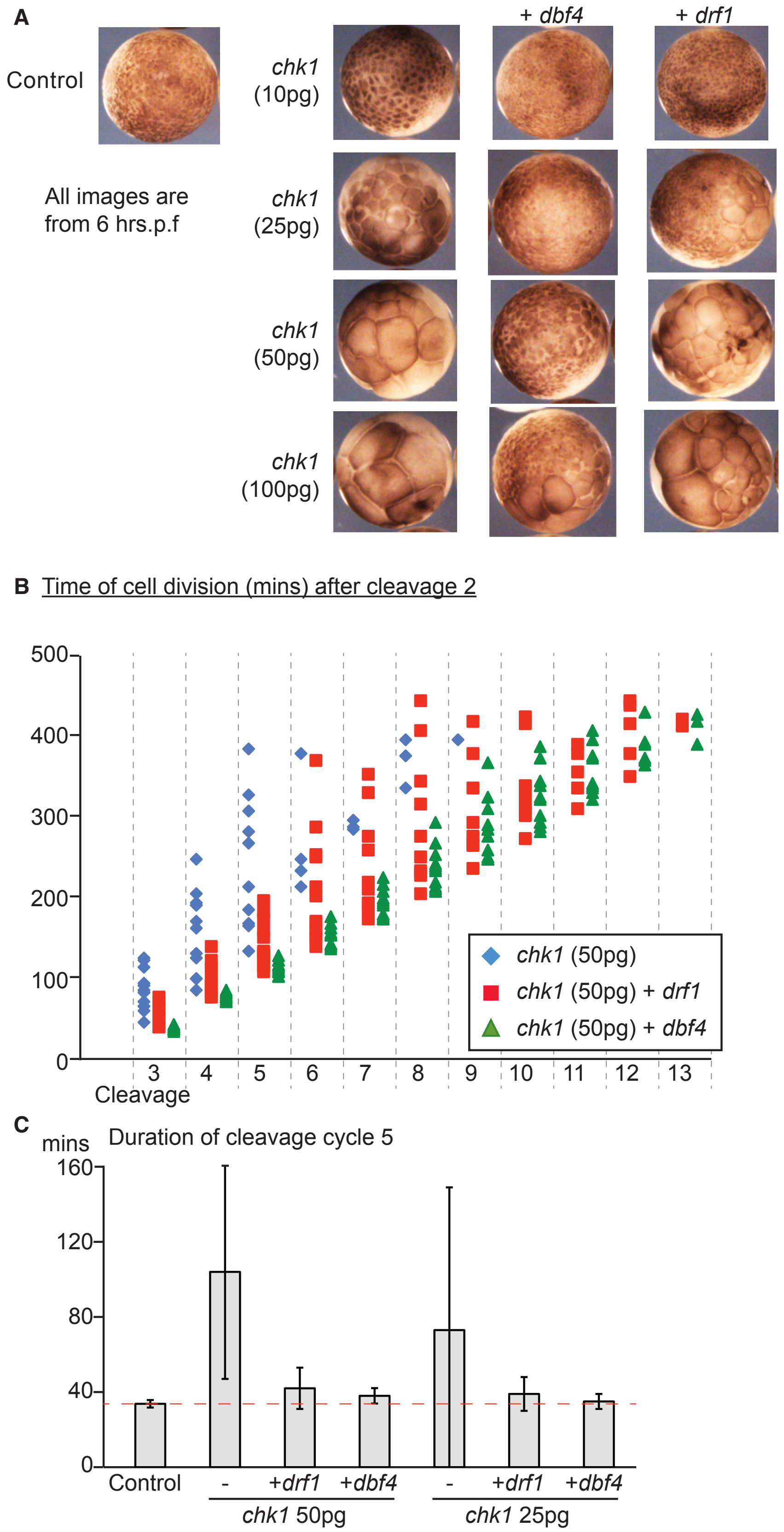

Figure 3. Chk1 Blocks the Cell Cycle by Inhibiting DDK

(A) Images of pre-MBT embryos (6 hr post fertilization), not expressing (Control) or expressing increasing amounts of *chk1* mRNA (pg), with or without co-expression of 500 pg of *drf1* or *dbf4*. See also Figures S1C–S1E.

(B) Analysis of the division of individual cells, as in Figure 1C, from movies of embryos expressing 50 pg of *chk1* mRNA, with or without co-expression of 500 pg of *drf1* or *dbf4*. The second division, generating the 4-cell embryo, was set to time zero. n = 12 cells from three embryos for each condition. See also Movie S5.

(C) The average duration of cell cycle 5, generating the 32-cell embryo. Red dashed line shows mean time of cycle 5 for control embryos. n = 12 cells from three embryos for each condition. Data are presented as mean ± SD, which indicates the level of synchrony of cell division. See also Figure S4.

Inhibitory Phosphorylation of Cdk1 Is Not Important for Cell-Cycle Lengthening at the MBT in *Xenopus*

In many organisms Chk1 inhibits cell-cycle progression by promoting the inhibitory phosphorylation of Cdk1, either by inactivating Cdc25 or activating Wee1 (Yuan et al., 2016). Indeed, this is an important mechanism that controls cell-cycle length during the MBT in *Drosophila* (Yuan et al., 2016). Since over-expression of Drf1 or Dbf4 is sufficient to suppress the cell-cycle delay caused by ectopic Chk1 expression (Figure 3C), we wondered whether the inhibitory phosphorylation of Cdk1 (also called Cdc2 in *Xenopus*) plays any role in Chk1-mediated control of cell-cycle progression in *Xenopus*. To explore this, we used a mutant of Cdk1 (*cdk1-AF*) that cannot be inhibited by Wee1/Myt1 phosphorylation because the inhibitory phosphorylation sites threonine 14 and tyrosine 15 are mutated to alanine and phenylalanine, respectively (Pickham et al., 1992). In contrast to Dbf4/Drf1 over-expression (Figure 3C), the *cdk1-AF* mutant had no effect on the Chk1-dependent block to cell-cycle progression (Figure S4). This observation is consistent with the inhibition of DDK being the primary mechanism for Chk1-mediated control of cell-cycle progression in the early *Xenopus* embryo (Figure 3).

Although Cdk1-AF over-expression could not suppress ectopic Chk1 activation, we wondered whether inhibitory phosphorylation of Cdk1 influences the elongation of the cell cycle at the MBT. Over-expression of *cdk1-AF* had no effect on cell-cycle lengthening during the MBT in *Xenopus* embryos (Figures S5A–S5C). Since one of the functions of Chk1 is to ensure the downregulation of Drf1, which is limiting at the MBT (Figure 2), *cdk1-AF* over-expression alone may not be sufficient to sustain rapid divisions at the MBT. Despite this, additional over-expression of Drf1 together with *cdk1-AF* still did not affect cell-cycle lengthening at the MBT (Figures S5D–S5F). Thus we find no evidence for a role for inhibitory phosphorylation of Cdk1 in regulating cell-cycle changes at the MBT in *X. laevis* (see Discussion).

SCF$^{\beta\text{-TRCP}}$ Regulates Drf1 Levels at the MBT

Given the significance of the Chk1-dependent downregulation of Drf1 at the MBT for control of the cell cycle (Figures 2 and 3), we set out to determine the mechanism for this regulation. Previous studies in *Xenopus* embryos have shown that Chk1 mediates the degradation of the CDK-activating phosphatase Cdc25A after the MBT (Shimuta et al., 2002) and that this requires the E3 ubiquitin ligase SCF$^{\beta\text{-TRCP}}$ (Kanemori et al., 2005). We therefore wondered whether a similar pathway might be responsible for the degradation of Drf1 at the MBT.

To test a role for SCF$^{\beta\text{-TRCP}}$ in Drf1 degradation, we modified the levels of the critical substrate recognition subunit β-Trcp in *Xenopus* embryos and analyzed the stability of Drf1 at the MBT. Injection of an anti-*β-trcp* antisense morpholino oligonucleotide reduced the levels of endogenous β-Trcp (Figure 4A, right) and resulted in the stabilization of Drf1 at the MBT (Figure 4A, left). Conversely, over-expression of β-Trcp caused even more rapid degradation of Drf1 (Figure 4B). Together these results suggest that, as with Cdc25, SCF$^{\beta\text{-TRCP}}$ regulates Drf1 at the MBT in *Xenopus*.

Stabilization of Drf1 (through inhibition of Chk1) delays it becoming limiting for S-phase progression at the MBT (Figure 2). To test the importance of SCF$^{\beta\text{-TRCP}}$-dependent degradation of Drf1 in ensuring that the cell cycle elongates at the MBT, we wondered whether over-expression of the other three limiting replication factors together with a reduction in SCF$^{\beta\text{-TRCP}}$ activity would be sufficient to drive fast cell cycles at the MBT. On its own, downregulation of *β-trcp* did not affect cell-cycle progression at the MBT (Movie S4 and Figures 4C–4E), and these embryos resembled the controls. However, when we over-expressed the remaining limiting replication factors, Treslin, Recq4, and Cut5, together with the *β-trcp* morpholino, this resulted in an extra division after the MBT (Figures 4D and 4E), generating embryos with more and smaller cells (Figure 4C). Conversely, when we over-expressed β-Trcp to induce earlier Drf1 degradation, we observed that the cell cycle was prematurely elongated (Figure S6). Together these results show that SCF$^{\beta\text{-TRCP}}$ regulates the levels of a critical limiting replication factor, Drf1, which can control cell-cycle duration in the early *Xenopus* embryo.

We have previously shown that Drf1, together with Cut5, Treslin and Recq4, are out-titrated on chromatin by increasing N/C ratios *in vitro*. Since Chk1 and SCF$^{\beta\text{-TRCP}}$ regulate Drf1 levels at the MBT *in vivo*, we wondered whether it is this pathway or the out-titration of Drf1 that causes this protein to become limiting at the MBT *in vivo*. By directly comparing SCF$^{\beta\text{-TRCP}}$ inhibition with Drf1 over-expression, we observed that stabilization of Drf1 levels, together with the over-expression of Cut5, Treslin, and Recq4, while sufficient for an extra division at the MBT, was not sufficient to reduce cell-cycle asynchrony during the MBT cycles 12 or 13 (Figure S7). On the other hand, over-expression of Drf1 plus the other three factors not only facilitated extra divisions but also caused these divisions to be rapid and synchronous (Figure S7). From this we conclude that stabilization of Drf1 at the MBT does not result in sufficient levels of Drf1 to overcome the increasing N/C ratio. Instead our data are consistent with a role for Drf1 degradation as a guarantee that the cell cycle elongates on time by ensuring that the levels of this protein are sufficiently low to be out-titrated by the increasing N/C ratio (see Discussion).

Chk1 Phosphorylates Drf1 for β-Trcp-Dependent Degradation

Since Chk1 and SCF$^{\beta\text{-TRCP}}$ both regulate Drf1 levels at the MBT *in vivo* (Figures 2 and 4), we set out to test how these pathways are connected. For Cdc25, Chk1-dependent phosphorylation of this protein generates a binding site for β-Trcp (which is an F-box protein), resulting in Cdc25 degradation (Kanemori et al., 2005). To address whether there is a similar phospho-dependent interaction between Drf1 and β-Trcp, we expressed tagged versions of both proteins in *Xenopus* embryos and analyzed their interaction in MBT-stage extracts by co-immunoprecipitation. Drf1, but not an unrelated protein of the same size (Smicl), immunoprecipitated β-Trcp from MBT-stage embryos (Figure 5A). Addition of a phosphatase to the extracts greatly reduced binding between Drf1 and β-Trcp, suggesting that this interaction is phospho-dependent (Figure 5B). Although we could not detect phosphorylated forms of full-length Drf1 on normal SDS-PAGE gels (e.g., Figure 5B), we did observe phosphorylated forms of Drf1 in MBT-stage embryos using Phos-tag PAGE gels (Figure 5C). Together these

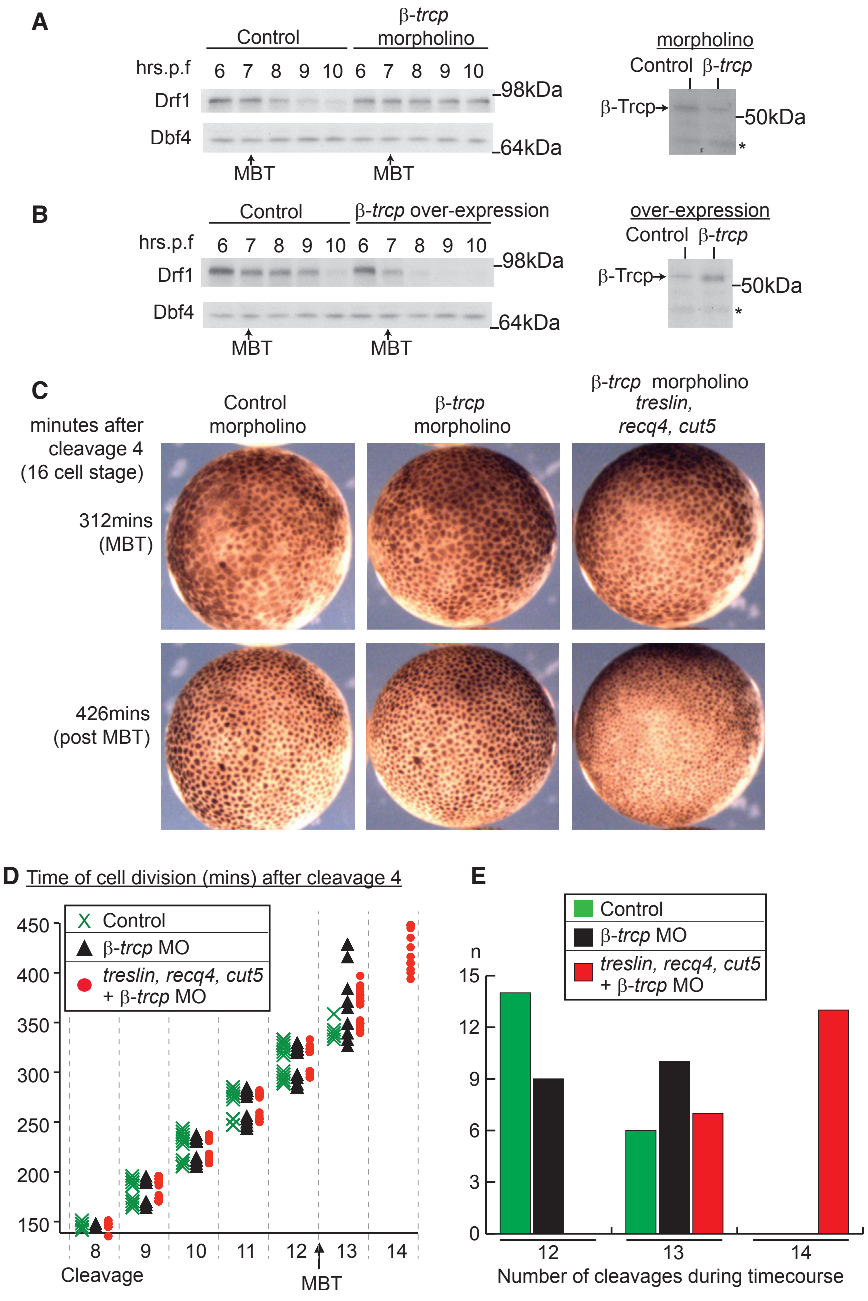

Figure 4. β-TRCP Controls Drf1 Levels

(A and B) Western blots as in Figure 2A (left for Drf1/Dbf4, right for β-TRCP at 8 hrs.p.f). For (A) the control was injection of a control morpholino. Asterisk denotes non-specific band.

(C–E) As for Figures 1B–1D. For (C) and (D), n = 20 cells from five embryos for each condition. MO, morpholino.

See also Movie S4 and Figure S6.

experiments demonstrate that phosphorylated Drf1 is bound by β-Trcp at the MBT.

β-Trcp binds substrates such as β-catenin through a characteristic interaction motif (DpSGΦXpS), where Φ represents a hydrophobic residue, X represents any amino acid, and pS represents phosphoserine (Silverman et al., 2012). We did not identify any perfect matches for this consensus sequence in *Xenopus* Drf1, so we expressed truncated forms of Drf1 to narrow down which regions might be required for β-Trcp binding and degradation. While a C-terminal fragment of Drf1 (453–784) remained stable, an N-terminal fragment (13–467) was degraded at the MBT like the full-length protein (Figure 5D). Importantly, this smaller N-terminal fragment of Drf1 exhibited a mobility shift from the MBT onward (Figure 5D). To test whether this modification was Chk1 dependent, we analyzed the Drf1-N terminal fragment with or without expression of the *chk1* dominant-negative mutant. While Drf1 modification and degradation was coincident with Chk1 activation in control embryos, the Chk1 D148A mutant prevented both the degradation and the appearance of lower-mobility forms of Drf1 (Figure 5E). We conclude from Figures 5D and 5E that Chk1-dependent phosphorylation and degron motifs are contained within the N-terminal region of Drf1.

Cdc25A, which is degraded in a Chk1 and β-Trcp-dependent manner in *Xenopus*, also lacks canonical β-Trcp interaction motifs (Kanemori et al., 2005), so we searched the Drf1 N terminus for degenerate β-Trcp binding sites. We identified two motifs with the consensus $DSX_{3\text{-}5}S$ in the Drf1 region 13–467 (Figure 5F). To test the role of these motifs in β-Trcp interaction and degradation, we deleted them and expressed the mutated *drf1* mRNA in *Xenopus* embryos. While full-length Drf1 interacted with β-Trcp and was degraded as expected at the time of the MBT, Drf1 lacking both $DSX_{3\text{-}5}S$ motifs (Δ) did not interact with β-Trcp (Figure 5G, left) and was stable at the MBT (Figure 5G, right). In addition to this truncation mutant we also generated a mutant of *drf1* where several key residues within the $DSX_{3\text{-}5}S$ motifs (asterisk in Figure 5F) were mutated to alanine (6A). As with the mutant lacking both motifs, the Drf1 6A mutant did not bind to β-Trcp and was not degraded (Figure 5H). To address whether Drf1 6A could still be phosphorylated by Chk1, we analyzed the phospho-shift of the N-terminal fragment of the protein. As expected, the Drf1 6A 13–467 N-terminal fragment was not degraded at the MBT, and although this protein still exhibited some mobility shift coincident with Chk1 activation, the ratio of phospho-Drf1 to unmodified Drf1 was less than for the wild-type protein (Figure 5I). Together, Figure 5 shows that Chk1

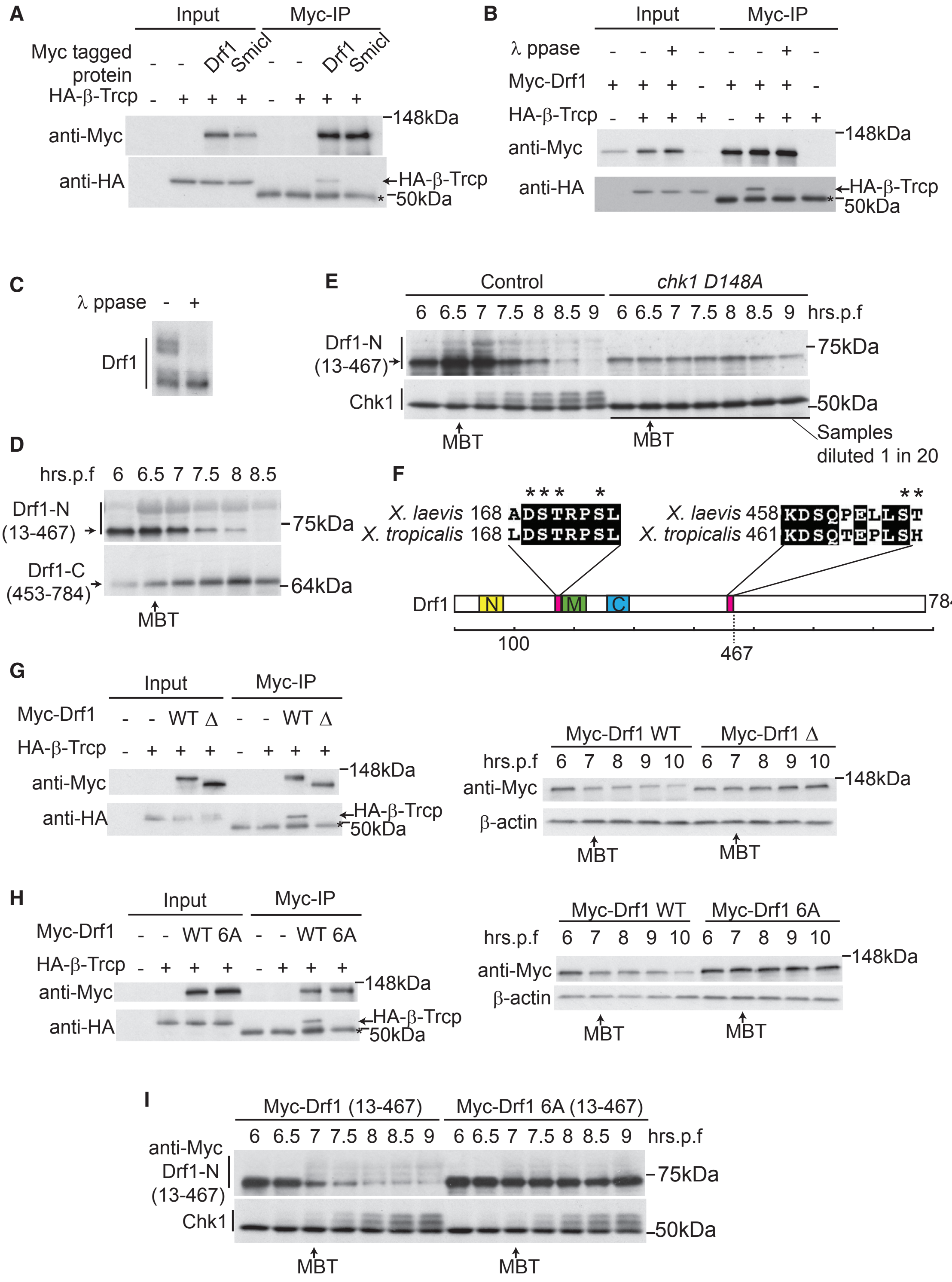

Figure 5. Chk1 Phosphorylates Drf1 for β-TRCP-Dependent Degradation

(A and B) Western blots after immunoprecipitation (IP) of myc-tagged Drf1 or unrelated myc-tagged protein of the same size (Smicl) from MBT-stage extracts co-expressing HA-tagged β-TRCP. Asterisk denotes immunoglobulin heavy chain.

(C) Anti-myc Western blot of myc-tagged Drf1 from MBT-stage embryos resolved on a phos-tag SDS-PAGE gel.

(D) Anti-myc Western blot of myc-tagged Drf1 fragments expressed in embryos and harvested at the indicated times.

(E) As in (D). For only the Chk1 blot from the over-expression of *chk1 D148A*, extracts were diluted 1 in 20 to allow a direct comparison between endogenous and over-expressed Chk1. All other western blots are of undiluted samples.

(legend continued on next page)

causes Drf1 phosphorylation and degradation, likely by generating a phospho-interaction between Drf1 and $SCF^{\beta\text{-}TRCP}$.

Downregulation of Drf1 Is Important for Embryogenesis

We have previously shown that only one replication factor needs to be limiting for the lengthening of the cell cycle at the MBT (Collart et al., 2013). As a consequence, while inhibition of Drf1 can act as a failsafe to guarantee the lengthening of the cell cycle at the MBT (see Discussion), over-expression of Drf1 alone (or inhibition of Chk1 or $SCF^{\beta\text{-}TRCP}$ alone) has little effect on the cell cycle during divisions 12–15 (Figures 2 and 4; Collart et al., 2013) because the other three factors (Treslin, Cut5, and Recq4) remain limiting. We therefore wondered whether there might be a role for Chk1-dependent downregulation of Drf1 in normal *Xenopus* development that is independent of cell-cycle control at the MBT.

Over-expression of Drf1 or the Drf1 6A mutant that is not degraded at the MBT (Figure 5) resulted in relatively normal development until gastrulation, at which point greater than 70% of embryos were still viable (stage 11, Figure 6A). However, these embryos exhibited a dramatic drop in viability during neurulation, and embryos expressing the Drf1 6A mutant failed to form neural folds (stage 20, Figure 6A). While a small fraction of Drf1 over-expressing embryos reached the tailbud stage (stage 32), none of the embryos expressing Drf1 6A were viable beyond this point in development (Figure 6A). These observations contrast dramatically with the over-expression of the Drf1 paralog Dbf4, whereby embryos remained fairly normal, with high viability during these embryonic stages (Figure 6A). This experiment shows that control of Drf1 levels, through Chk1- and $SCF^{\beta\text{-}TRCP}$-dependent degradation, is important for early vertebrate development.

Inhibition of Chk1 through over-expression of the *chk1 D148A* dominant-negative mutant results in the onset of cell death and loss of viability during gastrulation, as previously described (Shimuta et al., 2002). As the phenotype of Drf1 over-expression (Figure 6A) is less severe than the phenotype after over-expression of the *chk1 D148A* dominant-negative mutant, we hypothesized that Chk1 must have other functions apart from the downregulation of Drf1.

Although we did not detect any role for Chk1-mediated regulation of Cdk1 for cell-cycle control during the MBT (Figure S5), we wondered whether both Drf1 downregulation and Cdk1 downregulation might be essential events during *Xenopus* embryogenesis. If this were the case we would expect deregulation of Drf1 and Cdk1 to have synergistic effects on viability during embryogenesis. While over-expression of *cdk1-AF* resulted in embryonic death during gastrulation at the end of stage 11, over-expression of both *drf1-6A* and *cdk1-AF* caused a more severe phenotype, with embryos dying at the blastula stage (Figure 6B). This shows that the regulation of both Drf1 and Cdk1 are important events during blastula-to-gastrula development in *X. laevis*.

Inhibition of Drf1 Is an Essential Function of Chk1

Chk1 is essential for embryogenesis, as embryos expressing a dominant-negative mutant of *chk1* all die during gastrulation (Shimuta et al., 2002). In addition, a mutant of Drf1 that is refractory to downregulation by Chk1 (Drf1-6A) is also lethal (Figure 6A). We hypothesized that if the downregulation of Drf1 is a critical function of Chk1, inhibition of Drf1 might at least partially rescue loss of Chk1 activity. To test this idea we downregulated Drf1 expression using *drf1* antisense morpholinos (Figure 7C). Partial inhibition of Drf1 affected embryonic development only slightly, while embryos expressing *chk1 D148A* all died at stage 11 (Figure 7A). Importantly, reducing Drf1 levels with an antisense morpholino partially rescued the embryonic death observed in the *chk1 D148A* mutant embryos, as these embryos survived until stage 12 (Figure 7A). We analyzed this rescue in detail by timing the onset of cell death in embryos after the MBT (Figure 7B). While over-expression of *chk1 D148A* resulted in cell death by stage 10.5, the *drf1* morpholino maintained viability in these embryos until entry into stage 12 (Figure 7B). This demonstrates that Drf1 inhibition is a critical function of Chk1 during early embryogenesis. Since *drf1* morpholinos can only partially rescue the loss of Chk1 function, other roles of Chk1 are likely to also be important during early embryogenesis, such as the regulation of Cdk1 (Figure 6B).

Although Chk1 inhibition alone has no detectable effect on S-phase length or cell-cycle control during the MBT (Figures 1 and 2), we wondered whether the *drf1* morpholino might rescue the *chk1 D148A* mutant phenotype because it partially inhibits DNA replication. To examine this we tested whether morpholinos against *cdc6* (Collart et al., 2013), an upstream component in DNA replication control, could also suppress the phenotypes of *chk1 D148A*. Unlike the *drf1* morpholinos, partial inhibition of Cdc6 did not rescue the *chk1 D148A* phenotype (Figure 7A). From this we conclude that Chk1-dependent inhibition of Drf1 is essential during early development, but not through its role in controlling the rates of replication initiation (see Discussion).

Together, these data demonstrate that Chk1-dependent inhibition of Drf1 is an essential function of Chk1 during *Xenopus* embryogenesis and is the mechanism by which Chk1 regulates cell-cycle progression in the early embryo.

DISCUSSION

Ensuring Cell-Cycle Elongation at the MBT

Proliferation control and cell-cycle remodeling are key features of embryonic development across organisms (Budirahardja and Gonczy, 2009). We have previously shown that changes in replication initiation and subsequently in S-phase length cause slowing of the cell cycle at the MBT during cycles 12–15 in *X. laevis* (Collart et al., 2013). Four key limiting replication initiation factors—Drf1, Treslin, Recq4, and Cut5—govern the rate of replication initiation at this stage of development and their

(F) Scale diagram of *Xenopus laevis* Drf1, showing the three conserved Dbf4 domains (N, M, and C) and the degenerate potential β-TRCP binding domains (pink) within the region 1–467. Top: alignment of potential β-TRCP binding sites between *X. laevis* and *Xenopus tropicalis*. Asterisks denote residues mutated to alanine in the Drf1 6A mutant.

(G and H) Left: immunoprecipitations as in (A). Right: western blot as in (D) and (E). WT, wild-type. Δ indicates Drf1 with both potential β-TRCP binding sites deleted. 6A denotes full-length Drf1 with the six residues marked by asterisks in (F) mutated to alanine.

(I) As in (E).

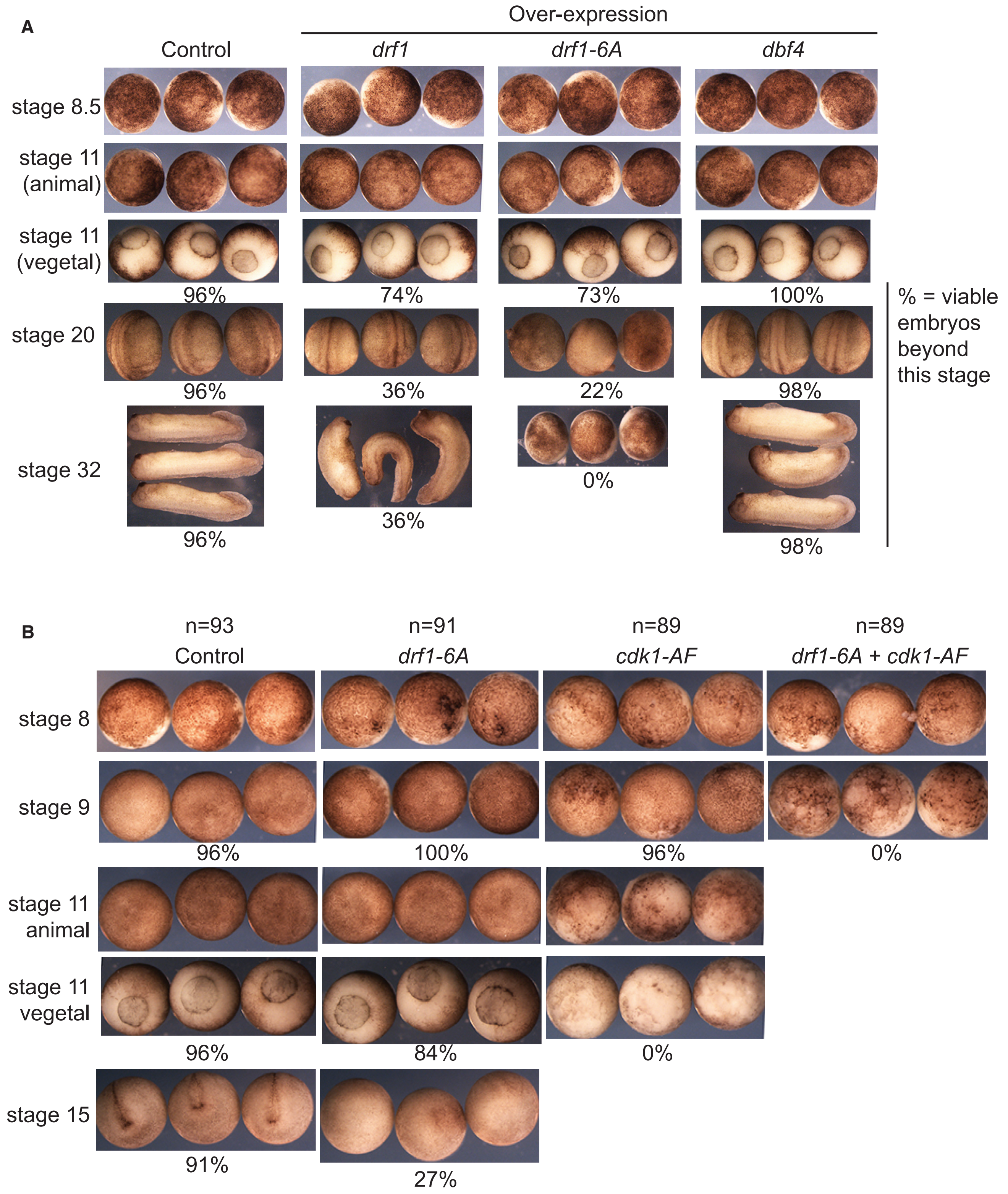

Figure 6. Inhibition of Drf1 Is Important for Development

(A) Images of staged embryos, injected at the 1-cell stage with water (control) or mRNA (over-expression). Percentages underneath the images represent the number of embryos that survived beyond that stage. n = 50 embryos for each condition.

(B) As in (A); 500 pg of *cdk1-AF* and 1 ng of *drf1* were injected at the 1-cell stage.

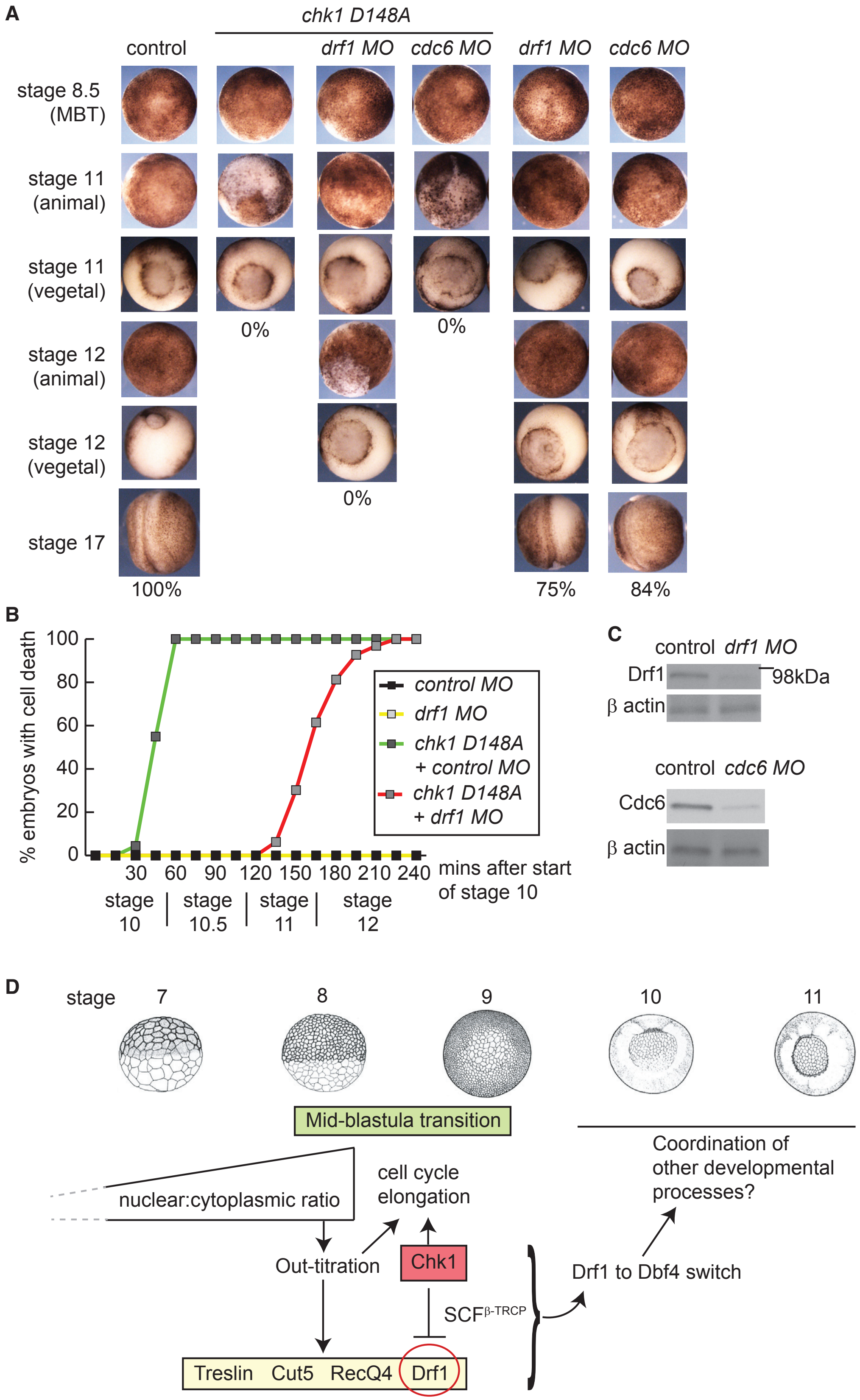

(legend on next page)

out-titration by the increasing N/C ratio acts as a timer for the elongation of the cell cycle at the MBT (Figure 7D). Despite this, how the precise amount of these proteins is regulated during early development is not known. Here we present a pathway that acts as a guarantee that the cell cycle elongates at the correct number of cell divisions (Figure 7D).

In addition to titrating key limiting replication factors (Collart et al., 2013), the N/C ratio is important for the developmental activation of Chk1 in *X. laevis* (Gotoh et al., 2011). We show here that a function of Chk1 at the MBT is to downregulate Drf1 through $SCF^{\beta\text{-}TRCP}$-dependent degradation (Figure 7D). As limiting the amount of any one of Drf1, Treslin, Recq4, or Cut5 is sufficient to elongate the cell cycle at the MBT, by degrading Drf1 this pathway guarantees that the cell cycle lengthens from cycle 12 onward, regardless of the levels of the other three factors (Figure 7D). Therefore, the N/C ratio serves as a robust timed switch for embryonic cell-cycle control both through out-titration of limiting replication factors and through Chk1 activation (Figure 7D).

Notably, we observe a difference in the cell cycle at the MBT depending on whether Drf1 is over-expressed or stabilized (e.g., by inhibition of $SCF^{\beta\text{-}TRCP}$). While over-expression of all four factors allows synchronous cleavages across cycles 12–15 (Figure 1), stabilization of Drf1 causes this protein to become limiting as early as cycle 13 (Figure S7). We conclude from this that Drf1 is limiting both by Chk1-$SCF^{\beta\text{-}TRCP}$-dependent degradation and by out-titration of this protein by the N/C ratio (Collart et al., 2013). Together, these mechanisms ensure that the cell cycle elongates in a timely manner at the MBT (Figure 7D).

A remaining question is how the N/C ratio causes Chk1 activation at the MBT in *Xenopus*. Work in *Drosophila* has suggested that conflict between replication and early zygotic transcription is a trigger for checkpoint activation (Blythe and Wieschaus, 2015), but it is unknown whether a similar mechanism exists in *Xenopus*. It is also unclear whether $SCF^{\beta\text{-}TRCP}$ is regulated during early embryogenesis, as the level of this ligase is also an important determinant of cell-cycle elongation (Figures 4 and S6).

Chk1 Regulation of DDK

Chk1 inhibits DNA replication both in cultured mammalian cells and in *Xenopus* egg extracts after replication stress and DNA damage (Heffernan et al., 2002; Maya-Mendoza et al., 2007; Platel et al., 2015). We have previously shown in budding yeast that the checkpoint kinase Rad53 inhibits replication initiation in part by targeting Dbf4 (Zegerman and Diffley, 2010). In this study we show that it is the Dbf4 ortholog Drf1 that is downregulated in a Chk1-dependent manner (Figures 2, 3, 4, and 5). DDK is clearly an important target of Chk1 in pre-MBT embryos because over-expression of either Dbf4 or Drf1 is sufficient to reverse the cell-cycle arrest caused by ectopic Chk1 over-expression (Figure 3). Since Drf1 and Dbf4 have equivalent roles in replication initiation (Silva et al., 2006; Takahashi and Walter, 2005) and are both able to suppress the Chk1-mediated arrest, we think it is likely that Chk1 blocks cell-cycle progression by inhibiting replication initiation. The ability of Drf1 over-expression to suppress the cell-cycle arrest induced by Chk1 (Figure 3) explains how over-expression of Drf1 (together with the other limiting replication factors) permits fast cell cycles at the MBT despite earlier Chk1 activation (Figure 1A).

It is intriguing that Drf1 is replaced by Dbf4 as the regulatory subunit of DDK kinase at the MBT (Silva et al., 2006; Takahashi and Walter, 2005). Our work suggests that the role of Chk1 in DNA replication control must also change after the MBT, when Drf1 is absent. It has been shown that Chk1 can bind to and inhibit Treslin to regulate replication initiation in human cells and *Xenopus* egg extracts (Guo et al., 2015). Our data show that Chk1 activation in pre-MBT embryos can be fully suppressed by over-expression of Dbf4 (Figure 3), suggesting that Treslin, which is essential for replication initiation, is not inhibited under these circumstances. In addition, Treslin is still limiting for rapid cell-cycle progression at the MBT even if Chk1 is inhibited (Figure S2). Although this suggests that Chk1 does not inhibit Treslin during embryogenesis, it does not preclude this as a mechanism for Chk1-dependent replication control, for example in somatic cells.

Chk1 Regulation of Cdk1

A well-established role of Chk1 both in the response to DNA damage (Bartek et al., 2004) and also during the MBT in *Drosophila* is to inhibit Cdk1 by either activating Wee1 or inhibiting Cdc25 (Yuan et al., 2016). Importantly, regulation of Cdk1 by Chk1 does not cause significant changes in cell cycles 12–15 in *Xenopus* either from the analysis of Cdc25 mutants (Shimuta et al., 2002) or from over-expression of an uninhibitable form of Cdk1 (*cdk1-AF*, Figure S5). In addition, inhibition of Wee1/Myt1 has little effect on cell-cycle duration in pre-MBT cycles (Tsai et al., 2014). Cdk1 regulation by Chk1 is also not a significant mechanism for cell-cycle arrest following ectopic expression of Chk1 in *Xenopus* embryos, because this is rescued solely by expression of Dbf4 or Drf1 (Figure 3) and not by the *cdk1-AF* allele (Figure S4).

Our data suggest that cell-cycle elongation at the MBT in *Xenopus* is controlled by changes in S-phase length throughout-titration and Chk1-dependent degradation of limiting replication factors (Figure 7D). This, however, does not exclude an important role for Cdk1 regulation from cycle 15 onward, and over-expression of the *cdk1-AF* mutant is indeed lethal after stage 11 (Figure 6). It is also striking that *Xenopus* Cdc25 regulation by Chk1 mirrors that of Drf1 (Figure 5), as Cdc25 is also inhibited by Chk1-dependent, $SCF^{\beta\text{-}TRCP}$-mediated degradation (Uto et al., 2004).

Figure 7. Inhibition of Drf1 Is a Crucial Function of Chk1

(A) As for Figures 6A and 6B. The control was injection of a control morpholino (MO). n = 50 embryos for each condition.

(B) Measurement of the appearance of cell death (white, extruded cells) from the start of stage 10 (9.5 hr post fertilization). n = 100 embryos for each condition.

(C) Western blot from stage-11 embryo extracts showing the partial knockdown of Drf1 and Cdc6 after the morpholino (MO) injections.

(D) The nuclear to cytoplasmic ratio ensures the lengthening of the cell cycle at the MBT both by out-titration of limiting replication factors (yellow box) and by inducing Chk1 activation, leading to Drf1 downregulation (red circle). See also Figure S7. Inhibition of Drf1 is the primary mechanism by which Chk1 elongates the cell cycle in the early embryo. Chk1-dependent degradation of Drf1 ensures a switch to Dbf4 as the regulatory subunit of DDK in post-MBT cycles. Downregulation of Drf1 is critical for developmental processes from the blastula stage onwards. Stages 1–6 are excluded for simplicity.

In *Drosophila* embryos, gradual changes in S-phase length in cycles 10–13 precede dramatic downregulation of Cdc25 (string and twine) in part caused by Chk1 (grapes) activation, resulting in even greater extension of S phase and the introduction of G_2 phase in cycle 14 (Farrell and O'Farrell, 2014; Farrell et al., 2012). Although extension of S phase is the initial cause of cell-cycle lengthening in both flies and frogs (Collart et al., 2013; Shermoen et al., 2010), a significant difference is that inhibition of Chk1 (grapes) in *Drosophila* is sufficient to shorten the cell cycle during the MBT cycles 11–13 (Sibon et al., 1997), which is not the case during the MBT cycles 12–15 in *Xenopus* (Figure 1). Further understanding of the functions of Chk1 in different organisms may help to explain such differences in embryonic cell-cycle control.

Drf1 Downregulation Is an Essential Function of Chk1

Chk1 is an essential enzyme during normal development in many metazoa (Fogarty et al., 1994; Kalogeropoulos et al., 2004; Liu et al., 2000; Shimuta et al., 2002; Takai et al., 2000). Indeed, inhibition of Chk1 leads to embryonic death during gastrulation in *X. laevis* (Figure 7A; Shimuta et al., 2002). We show here that downregulation of Drf1 is a critical function of Chk1 during early embryogenesis (Figure 6) and that inhibition of Drf1 partially rescues the *chk1 D148A* mutant phenotype (Figure 7). Since *drf1* morpholinos can only partially rescue the loss of Chk1, it is likely that Chk1 has other important functions during embryogenesis such as the regulation of Cdc25. Indeed the *cdk1-AF* mutant, which cannot be regulated by the Wee1/Cdc25 axis, shows synergistic lethality with *drf1-6A* (Figure 6B).

In normal embryos, Chk1 activation ensures that Drf1 is replaced by its paralog Dbf4 after the MBT (Figure 2; Silva et al., 2006; Takahashi and Walter, 2005). An important remaining question is why degradation of Drf1 is so important for the blastula to gastrula stage of development. Both Drf1 and Dbf4 have equivalent roles in replication initiation (Silva et al., 2006; Takahashi and Walter, 2005) and are both capable of driving replication initiation in MBT-stage embryos (data not shown). Several lines of evidence suggest that the essential role of Drf1 downregulation might not be due to shared functions with Dbf4 in replication initiation control. First, Drf1 over-expression alone, which is not sufficient to induce high replication initiation rates (Collart et al., 2013), is by itself lethal during early embryogenesis, whereas Dbf4 over-expression is not (Figure 6A). In addition, although downregulation of Drf1 partially rescues the Chk1 dominant-negative phenotype, inhibition of Cdc6, an upstream component of replication initiation, has no effect (Figure 7A). Therefore, while Drf1 downregulation by Chk1 acts as a guarantee that the cell cycle lengthens at the MBT, there must also be additional functions for a handover between Drf1 and Dbf4 at this stage in development (Figure 7D).

Although we cannot rule out the possibility that there are subtle consequences for cell division if Drf1 levels are high during the MBT, we hypothesize that Drf1 and Dbf4 may have functions beyond DNA replication that must be correctly timed during embryogenesis. Such functions would explain why Drf1 and Dbf4, although equivalent in their role in replication initiation, have such different expression patterns and regulation during embryogenesis (this study; Silva et al., 2006; Takahashi and Walter, 2005). Interestingly, Dbf4 has been suggested to be an inhibitor of Wnt-driven transcription (Brott and Sokol, 2005), which is switched on at the MBT in *Xenopus* (Hikasa and Sokol, 2013). This may not be a unique feature of Dbf4, however, because we observe similar phenotypes with Drf1, such as embryo ventralization after over-expression (e.g., Figure 6A). To understand the physiological roles of the Chk1-dependent switch from Drf1 to Dbf4 at the MBT, it will be important to further investigate the different functions of these proteins during embryogenesis.

Both Drf1 and Dbf4 orthologs exist in humans (Montagnoli et al., 2002), and Cdc7 kinase is emerging as a potential target in certain cancers (Huggett et al., 2016). In addition, Chk1 inhibitors are currently in clinical trials as chemotherapeutics (Puigvert et al., 2016). Understanding the different functions of Drf1 and Dbf4, together with their interplay with Chk1 in vertebrates, may have significant implications for the use and effectiveness of drugs that target these proteins in humans.

STAR★METHODS

Detailed methods are provided in the online version of this paper and include the following:

- KEY RESOURCES TABLE
- CONTACT FOR REAGENT AND RESOURCE SHARING
- EXPERIMENTAL MODEL AND SUBJECT DETAILS
  - *Xenopus laevis* Induction and Husbandry
  - *Xenopus laevis* Embryo Culture and Injections
- METHOD DETAILS
  - Cloning and *In Vitro* Transcription
  - Western Blotting
  - Co-Immunoprecipitation Experiments
  - Phostag Gel Electrophoresis
  - Preparation of Bulk Genomic DNA
- QUANTIFICATION AND STATISTICAL ANALYSIS
  - Movies

SUPPLEMENTAL INFORMATION

Supplemental Information includes seven figures and five movies and can be found with this article online at http://dx.doi.org/10.1016/j.devcel.2017.06.010.

AUTHOR CONTRIBUTIONS

C.C., P.Z., and J.C.S. conceived and designed the experiments, which were all performed by C.C. P.Z. wrote the paper.

ACKNOWLEDGMENTS

We are grateful to Tatsuro Takahashi (Osaka University) and Johannes Walter (Harvard Medical School) for *Xenopus* Drf1 and Dbf4 antibodies. We thank members of the P.Z. and J.C.S. laboratories for comments and critical reading of the manuscript. The work was supported by Worldwide Cancer Research 10-0908, Wellcome Trust 107056/Z/15/Z, Gurdon Institute funding (Cancer Research UK C6946/A14492, Wellcome Trust 092096), and Francis Crick Institute funding (Cancer Research UK FC001-157, the UK Medical Research Council FC001-157, Wellcome Trust FC001-157). We are grateful to Xenbase (www.xenbase.org) for the use of *Xenopus* embryo images in Figure 7D.

Received: August 2, 2016
Revised: April 19, 2017
Accepted: June 7, 2017
Published: July 10, 2017

REFERENCES

Bartek, J., Lukas, C., and Lukas, J. (2004). Checking on DNA damage in S phase. Nat. Rev. Mol. Cell Biol. *5*, 792–804.

Blythe, S.A., and Wieschaus, E.F. (2015). Zygotic genome activation triggers the DNA replication checkpoint at the midblastula transition. Cell *160*, 1169–1181.

Brott, B.K., and Sokol, S.Y. (2005). A vertebrate homolog of the cell cycle regulator Dbf4 is an inhibitor of Wnt signaling required for heart development. Dev. Cell *8*, 703–715.

Budirahardja, Y., and Gonczy, P. (2009). Coupling the cell cycle to development. Development *136*, 2861–2872.

Collart, C., Allen, G.E., Bradshaw, C.R., Smith, J.C., and Zegerman, P. (2013). Titration of four replication factors is essential for the *Xenopus laevis* midblastula transition. Science *341*, 893–896.

Farrell, J.A., and O'Farrell, P.H. (2014). From egg to gastrula: how the cell cycle is remodeled during the *Drosophila* mid-blastula transition. Annu. Rev. Genet. *48*, 269–294.

Farrell, J.A., Shermoen, A.W., Yuan, K., and O'Farrell, P.H. (2012). Embryonic onset of late replication requires Cdc25 down-regulation. Genes Dev. *26*, 714–725.

Ferree, P.L., Deneke, V.E., and Di Talia, S. (2016). Measuring time during early embryonic development. Semin. Cell Dev. Biol. *55*, 80–88.

Fogarty, P., Kalpin, R.F., and Sullivan, W. (1994). The *Drosophila* maternal-effect mutation grapes causes a metaphase arrest at nuclear cycle 13. Development *120*, 2131–2142.

Gotoh, T., Kishimoto, T., and Sible, J.C. (2011). Phosphorylation of Claspin is triggered by the nucleocytoplasmic ratio at the *Xenopus laevis* midblastula transition. Dev. Biol. *353*, 302–308.

Guo, C., Kumagai, A., Schlacher, K., Shevchenko, A., Shevchenko, A., and Dunphy, W.G. (2015). Interaction of Chk1 with Treslin negatively regulates the initiation of chromosomal DNA replication. Mol. Cell *57*, 492–505.

Heffernan, T.P., Simpson, D.A., Frank, A.R., Heinloth, A.N., Paules, R.S., Cordeiro-Stone, M., and Kaufmann, W.K. (2002). An ATR- and Chk1-dependent S checkpoint inhibits replicon initiation following UVC-induced DNA damage. Mol. Cell. Biol. *22*, 8552–8561.

Hensey, C., and Gautier, J. (1997). A developmental timer that regulates apoptosis at the onset of gastrulation. Mech. Dev. *69*, 183–195.

Hikasa, H., and Sokol, S.Y. (2013). Wnt signaling in vertebrate axis specification. Cold Spring Harb. Perspect. Biol. *5*, a007955.

Howe, J.A., and Newport, J.W. (1996). A developmental timer regulates degradation of cyclin E1 at the midblastula transition during *Xenopus* embryogenesis. Proc. Natl. Acad. Sci. USA *93*, 2060–2064.

Huggett, M.T., Tudzarova, S., Proctor, I., Loddo, M., Keane, M.G., Stoeber, K., Williams, G.H., and Pereira, S.P. (2016). Cdc7 is a potent anti-cancer target in pancreatic cancer due to abrogation of the DNA origin activation checkpoint. Oncotarget *7*, 18495–18507.

Hyrien, O., Maric, C., and Mechali, M. (1995). Transition in specification of embryonic metazoan DNA replication origins. Science *270*, 994–997.

Kalogeropoulos, N., Christoforou, C., Green, A.J., Gill, S., and Ashcroft, N.R. (2004). chk-1 is an essential gene and is required for an S-M checkpoint during early embryogenesis. Cell Cycle *3*, 1196–1200.

Kane, D.A., and Kimmel, C.B. (1993). The zebrafish midblastula transition. Development *119*, 447–456.

Kanemori, Y., Uto, K., and Sagata, N. (2005). Beta-TrCP recognizes a previously undescribed nonphosphorylated destruction motif in Cdc25A and Cdc25B phosphatases. Proc. Natl. Acad. Sci. USA *102*, 6279–6284.

Kappas, N.C., Savage, P., Chen, K.C., Walls, A.T., and Sible, J.C. (2000). Dissection of the XChk1 signaling pathway in *Xenopus laevis* embryos. Mol. Biol. Cell *11*, 3101–3108.

Kumagai, A., Shevchenko, A., and Dunphy, W.G. (2011). Direct regulation of Treslin by cyclin-dependent kinase is essential for the onset of DNA replication. J. Cell Biol. *193*, 995–1007.

Labib, K. (2010). How do Cdc7 and cyclin-dependent kinases trigger the initiation of chromosome replication in eukaryotic cells? Genes Dev. *24*, 1208–1219.

Langley, A.R., Smith, J.C., Stemple, D.L., and Harvey, S.A. (2014). New insights into the maternal to zygotic transition. Development *141*, 3834–3841.

Liu, Q., Guntuku, S., Cui, X.S., Matsuoka, S., Cortez, D., Tamai, K., Luo, G., Carattini-Rivera, S., DeMayo, F., Bradley, A., et al. (2000). Chk1 is an essential kinase that is regulated by Atr and required for the G(2)/M DNA damage checkpoint. Genes Dev. *14*, 1448–1459.

Maya-Mendoza, A., Petermann, E., Gillespie, D.A., Caldecott, K.W., and Jackson, D.A. (2007). Chk1 regulates the density of active replication origins during the vertebrate S phase. EMBO J. *26*, 2719–2731.

Montagnoli, A., Bosotti, R., Villa, F., Rialland, M., Brotherton, D., Mercurio, C., Berthelsen, J., and Santocanale, C. (2002). Drf1, a novel regulatory subunit for human Cdc7 kinase. EMBO J. *21*, 3171–3181.

Nakajo, N., Oe, T., Uto, K., and Sagata, N. (1999). Involvement of Chk1 kinase in prophase I arrest of *Xenopus* oocytes. Dev. Biol. *207*, 432–444.

Newport, J., and Dasso, M. (1989). On the coupling between DNA replication and mitosis. J. Cell Sci. Suppl. *12*, 149–160.

Newport, J., and Kirschner, M. (1982a). A major developmental transition in early *Xenopus* embryos: I. characterization and timing of cellular changes at the midblastula stage. Cell *30*, 675–686.

Newport, J., and Kirschner, M. (1982b). A major developmental transition in early *Xenopus* embryos: II. control of the onset of transcription. Cell *30*, 687–696.

Nieuwkoop, P.D., and Faber, J. (1994). Normal Table of *Xenopus laevis* (Daudin): A Systematical and Chronological Survey of the Development from the Fertilized Egg till the End of Metamorphosis (Garland Pub.).

Pickham, K.M., Meyer, A.N., Li, J., and Donoghue, D.J. (1992). Requirement of mosXe protein kinase for meiotic maturation of *Xenopus* oocytes induced by a cdc2 mutant lacking regulatory phosphorylation sites. Mol. Cell. Biol. *12*, 3192–3203.

Platel, M., Goldar, A., Wiggins, J.M., Barbosa, P., Libeau, P., Priam, P., Narassimprakash, H., Grodzenski, X., and Marheineke, K. (2015). Tight Chk1 levels control replication cluster activation in *Xenopus*. PLoS One *10*, e0129090.

Puigvert, J.C., Sanjiv, K., and Helleday, T. (2016). Targeting DNA repair, DNA metabolism and replication stress as anti-cancer strategies. FEBS J. *283*, 232–245.

Shermoen, A.W., McCleland, M.L., and O'Farrell, P.H. (2010). Developmental control of late replication and S phase length. Curr. Biol. *20*, 2067–2077.

Shimuta, K., Nakajo, N., Uto, K., Hayano, Y., Okazaki, K., and Sagata, N. (2002). Chk1 is activated transiently and targets Cdc25A for degradation at the *Xenopus* midblastula transition. EMBO J. *21*, 3694–3703.

Sibon, O.C., Stevenson, V.A., and Theurkauf, W.E. (1997). DNA-replication checkpoint control at the *Drosophila* midblastula transition. Nature *388*, 93–97.

Silva, T., Bradley, R.H., Gao, Y., and Coue, M. (2006). *Xenopus* CDC7/DRF1 complex is required for the initiation of DNA replication. J. Biol. Chem. *281*, 11569–11576.

Silverman, J.S., Skaar, J.R., and Pagano, M. (2012). SCF ubiquitin ligases in the maintenance of genome stability. Trends Biochem. Sci. *37*, 66–73.

Slack, J.M. (1984). Regional biosynthetic markers in the early amphibian embryo. J. Embryol. Exp. Morphol. *80*, 289–319.

Tadros, W., and Lipshitz, H.D. (2009). The maternal-to-zygotic transition: a play in two acts. Development *136*, 3033–3042.

Takahashi, T.S., and Walter, J.C. (2005). Cdc7-Drf1 is a developmentally regulated protein kinase required for the initiation of vertebrate DNA replication. Genes Dev. *19*, 2295–2300.

Takai, H., Tominaga, K., Motoyama, N., Minamishima, Y.A., Nagahama, H., Tsukiyama, T., Ikeda, K., Nakayama, K., Nakanishi, M., and Nakayama, K. (2000). Aberrant cell cycle checkpoint function and early embryonic death in Chk1(-/-) mice. Genes Dev. *14*, 1439–1447.

Tsai, T.Y., Theriot, J.A., and Ferrell, J.E., Jr. (2014). Changes in oscillatory dynamics in the cell cycle of early *Xenopus laevis* embryos. PLoS Biol. *12*, e1001788.

Uto, K., Inoue, D., Shimuta, K., Nakajo, N., and Sagata, N. (2004). Chk1, but not Chk2, inhibits Cdc25 phosphatases by a novel common mechanism. EMBO J. *23*, 3386–3396.

Yuan, K., Seller, C.A., Shermoen, A.W., and O'Farrell, P.H. (2016). Timing the drosophila mid-blastula transition: a cell cycle-centered view. Trends Genet. *32*, 496–507.

Zegerman, P., and Diffley, J.F. (2010). Checkpoint-dependent inhibition of DNA replication initiation by Sld3 and Dbf4 phosphorylation. Nature *467*, 474–478.

STAR★METHODS

KEY RESOURCES TABLE

REAGENT or RESOURCE	SOURCE	IDENTIFIER
Antibodies		
Polyclonal rabbit anti-Drf1 antibody	Gift from Tatsuro Takahashi (Osaka University) and Johannes Walter (Harvard Medical School)	(Takahashi and Walter, 2005)
Polyclonal rabbit anti-Dbf4 antibody	Gift from Tatsuro Takahashi (Osaka University) and Johannes Walter (Harvard Medical School)	(Takahashi and Walter, 2005)
Mouse monoclonal anti-Myc antibody (lyophilized) and dissolved in water to a concentration of 1 mg/ml	Roche	(cat # 11667149001)
Mouse monoclonal anti-HA antibody	Origene	(cat # TA180128)
Mouse monoclonal anti-Chk1 antibody (DCS-310)	Thermo Scientific	(cat # MA1-91087)
Rabbit polyclonal anti-βTrcp	Abcam	(ab137674)
Mouse monoclonal anti-Myc antibody (9E10) coated magnetic beads	Origene	(cat # TA150044)
Biological Samples		
Xenopus laevis embryos	NASCO	N/A
Chemicals, Peptides, and Recombinant Proteins		
Phos-tag acrylamide	Alpha Laboratories Ltd	(cat #304-93521)
Trizol reagent	Invitrogen	(cat #15596-026)
Critical Commercial Assays		
Transcriptor First Strand cDNA kit	Roche	(cat #04379012001)
mMESSAGE mMACHINE SP6 Transcription Kit	ThermoFisher Scientific	AM1340
Aphidicolin	Sigma	Cat 0781
Experimental Models: Organisms/Strains		
Xenopus laevis WT	NASCO	N/A
Oligonucleotides		
Morpholino<br>*cdc6A*MO:<br>5'-CTGGTGCTTGGCATGGCTGCTTGTC-3'<br>*cdc6B*MO:<br>5'-AATTCAGTCAGAAATAACCAGGCTC-3'	(Collart et al., 2013)<br>Gene Tools	N/A
*drf1*MO:<br>5'-GCAGAACAGAGATCACACTGGCCAT-3'	(Collart et al., 2013)<br>Gene Tools	N/A
*βtrcp*MO:<br>5'-GAGAACATGAAAATCCTTCCATCTC-3'	This paper<br>Gene Tools	N/A
*dbf4*MO:<br>5′-CACTGCTGCTATGGTAGATTTCATT-3′	Described in (Brott and Sokol, 2005) as XDMO1<br>Gene Tools	N/A
coMO:<br>5'-CCTCTTACCTCAGTTACAATTTATA 3'	Standard control morpholino from Gene Tools	N/A
Recombinant DNA		
Xenopus laevis chk1 in pCS2	This paper	N/A
Xenopus laevis chk1 D148A in pCS2	This paper	N/A
Xenopus laevis β-trcp in pCS2 with N-terminal HA tag	This paper	N/A
Xenopus laevis dbf4 in PCS2	This paper	N/A
Xenopus laevis drf1 (Drf1) with N-terminal 6xMyc tag in PCS2	This paper	N/A
Xenopus laevis drf1 (Drf1-N) with N-terminal 6xMyc tag in pCS2	This paper	N/A
Xenopus laevis drf1 with 6 amino acid substitutions (Drf1 6A) with N-terminal 6xMyc tag in pCS2	This paper	N/A

(*Continued on next page*)

Continued

REAGENT or RESOURCE	SOURCE	IDENTIFIER
Xenopus laevis drf1 (13-467) with 6 amino acid substitutions and N-terminal 6xMyc tag in pCS2 (Drf1 6A)	This paper	N/A
Xenopus laevis cdk1 in pCS2 with N-terminal HA tag	This paper	N/A
Xenopus laevis cdk1AF in pCS2 with N-terminal HA tag	(Pickham et al., 1992)	N/A
Xenopus laevis drf1 lacking amino acids 165-174 and amino acids 455-467 (Drf1 Δ) with N-terminal 6xMyc tag in pCS2	This paper	N/A
Xenopus laevis treslin in pCS2 with C-terminal Flag tag	(Collart et al., 2013)	N/A
Xenopus laevis recq4 in pCS2	(Collart et al., 2013)	N/A
Xenopus laevis drf1 in pCITE4a	Gift from Tatsuro Takahashi (Osaka University, Japan)(Collart et al., 2013)	N/A
Xenopus laevis cut5 in pCS2	Gift from Haruhiko Takisawa (Osaka University, Japan)	N/A
Software and Algorithms		
Fiji	N/A	N/A

CONTACT FOR REAGENT AND RESOURCE SHARING

Further information and requests for resources and reagents should be directed to and will be fulfilled by the Lead Contact, Philip Zegerman (paz20@cam.ac.uk).

EXPERIMENTAL MODEL AND SUBJECT DETAILS

Xenopus laevis Induction and Husbandry

Regulations for the use of *Xenopus laevis*, as outlined in the Animals Scientific Procedures Act (ASPA) and implemented by the Home Office in the UK, were followed.

Frogs were obtained from NASCO and kept in a *Xenopus* research facility designed by Aqua Schwarz. Tanks contained dechlorinated water at 19°C, pH 7.5 and conductivity between 900-1100 μS/cm. Ammonia and nitrite levels were at 0 ppm. The light/dark cycle in the room was 12h/12h. Tanks were populated with 3 females or 6 males. Frogs were fed a diet of 3 x 1 g of 5LP3 frog diet advanced protocol (from labdiet), per frog and per week.

Females were induced by injection of 400 U Chorulon (Human Chorionic Gonadotrophin), followed by injection of 50 U PMSG (Pregnant Mare's Serum Gonadotrophin) from Intervet within 10 to 12 days. Males were killed by injection of an overdose (160 mg) of the anesthetic MS222 dissolved in water.

Adult males and females were only used to obtain sperm and eggs respectively.

All experiments were performed on embryos.

Xenopus laevis Embryo Culture and Injections

Embryos of *Xenopus laevis* were obtained by artificial fertilisation. They were maintained in 10% normal amphibian medium (NAM) (100 mM NaCl, 2 mM KCl, 1 mM $Ca(NO_3)_2.4H_2O$, 1 mM $MgSO_4$.7H2O, 0.1mMEDTA, 0.02 mM NaH_2PO_4.2H2O, 0.08 mM $Na_2HPO_4.2H_2O$) (Slack, 1984) at 20 °C and staged (Nieuwkoop and Faber, 1994). *Xenopus* embryos were injected at the one or two cell stage (as indicated in the figure legends) with antisense morpholino oligonucleotides (MOs) (dissolved in water) obtained from GeneTools, LLC or with sense RNA obtained by in vitro transcription (as indicated in the figure legends).

METHOD DETAILS

Cloning and *In Vitro* Transcription

Total RNA was isolated from *Xenopus* embryos using the TriPure reagent (Invitrogen), followed by a LiCl precipitation. cDNA was prepared by reverse transcription, using the Transcriptor First Strand cDNA kit (Roche).

The coding sequence of *Xenopus laevis chk1* was amplified by PCR and cloned between the EcoRI and XhoI sites of pCS2. cDNA encoding a dominant negative Asp148 to Ala mutant (Chk1D148A, Nakajo et al., 1999), was cloned between the EcoRI and XhoI sites of pCS2 . The coding sequence of *Xenopus laevis β-trcp* was amplified by PCR and cloned in frame with an N-terminal HA tag between the ClaI and XhoI sites of pCS2.

The coding sequence of *Xenopus laevis dbf4* was amplified by PCR and cloned between the EcoRI and XhoI sites of pCS2. Drf1 full length and N and C truncations were amplified by PCR and cloned between the EcoRI and XhoI sites of Myc-pCS2. Drf1 Δ corresponds to *drf1* lacking amino acids 165-174 and amino acids 455-467. cDNA encoding *drf1* with 6 amino acid substitutions to alanine

(Drf1 6A) at the following positions (Asp169, Ser170, Thr171, Ser174, Ser466 and Thr467) was cloned between the EcoRI and XhoI sites of Myc-pCS2. The coding sequence of *Xenopus laevis cdk1* (Cdk1) and Cdk1AF (T15A, Y16F - Pickham et al., 1992) were amplified by PCR and cloned in frame with an N-terminal HA tag. Amino acids 215-474 of *Xenopus laevis Chk1* (Myc-Chk1ΔKD) was amplified by PCR and cloned between the EcoRI and XhoI sites of Myc-pCS2.

To obtain sense RNA from these constructs, plasmids were digested with Not1 followed by in vitro transcription using SP6 RNA polymerase.

To obtain sense RNA, *treslin*, *cut5*, *drf1* and *recq4* containing plasmids (described in (Collart et al., 2013)) were digested respectively with Not1, Asp718, SpeI and Asp718. SP6 RNA polymerase was used for in vitro transcription of *treslin*, *cut5* and *recq4* and T7 polymerase for *drf1*.

Western Blotting

Anti-Drf1 and anti-Dbf4 were used at concentrations of 1/10000 and 1/3000 respectively in PBS with 0.1% Tween and incubated overnight. at 4°C. Anti-HA, anti-Myc and anti-βTrcp were used at a concentration of 1/1000 in PBS+0.1%Tween and incubated 1 hr at room temperature. Anti-Chk1 was used at a concentration of 1/100 in PBS+0.1%Tween and incubated 1 hr at RT. Goat anti-rabbit (Thermo Scientific 31466) and horse anti-mouse (Vector Laboratories PI 2000) HRP coupled antibodies were used as secondary antibodies and used at concentrations of 1/10000 in PBS+0.1%Tween and incubated for 1 hour at room temperature.

Co-Immunoprecipitation Experiments

Xenopus embryos were frozen in liquid nitrogen and solubilised in lysis buffer containing 1% NP40, 150 mM NaCl, 20 mM Tris pH 7.5, 2 mM EDTA, 50 mM NaF, 1 mM sodium pyrophosphate, supplemented with protease inhibitors (Roche). Yolk and lipids were extracted with Freon and lysates were cleared by centrifugation. Precipitations were performed by overnight incubations with mouse monoclonal anti-Myc coated magnetic beads at 4°C. Unbound proteins were removed by washing four times with lysis buffer and once with phosphate-buffered saline at 4°C. Bound proteins were harvested by boiling in sample buffer, and they were resolved by SDS-polyacrylamide gel electrophoresis.

Phostag Gel Electrophoresis

Xenopus embryos were frozen in liquid nitrogen and solubilised in lysis buffer containing 1% NP40, 150 mM NaCl, 20 mM Tris pH 7.5 and 2 mM EDTA, supplemented with protease inhibitors (Roche). Yolk and lipids were extracted with Freon and lysates were cleared by centrifugation. Proteins were precipitated with trichloroacetic acid (final concentration 12.5%). After washing the pellet in ice-cold acetone, proteins were dissolved in 1x restriction enzyme buffer 3 from NEB. $MnCl_2$ was added to a final concentration of 0.066 mM. Proteins were then resolved by SDS polyacrylamide gel electrophoresis with resolving gel – 4% acrylamide, acrylamide/bisacrylamide = 29/1, 0.5% agarose, 0.035 mM $MnCl_2$, 375 mM Tris pH 8.8, 0.1% SDS, 0.001 % Temed, 0.05% APS, 0.0125 mM Phos-tag acrylamide (Alpha Laboratories Ltd) and stacking gel – 3% acrylamide, acrylamide/bisacrylamide = 29/1, 125 mM Tris pH 6.8, 0.001% Temed, 0.05% APS. The gel was incubated 3 X 15 min in 10 mM EDTA before Western blotting.

Preparation of Bulk Genomic DNA

Xenopus embryos were dissolved in lysis buffer (150 mM NaCl, 10 mM EDTA, 50 mM Tris pH 7.5, 0.5 % SDS) and treated with RNAse A for 2 hours at 37°C (final concentration 20 μg/ml) and then overnight with proteinase K at 55°C (final concentration 0.25 mg/ml). After phenol/chloroform extraction and ethanol precipitation of the sample, DNA pellets were dissolved in H_2O and loaded onto a 1% agarose gel.

QUANTIFICATION AND STATISTICAL ANALYSIS

Movies

Movies were made with a Leica MZ FL III microscope at 20°C and analysed with ImageJ (Fiji) software. Numbers of cells and embryos analysed are indicated in the figure legends.

Numbers of embryos injected for phenotypical analysis are indicated in the figures and figure legends.

The quantification of bulk genomic DNA was performed on three biological replicates and the intensity of the bands was scanned and analysed with ImageJ software.

Developmental Cell, Volume *42*

Supplemental Information

Chk1 Inhibition of the Replication Factor Drf1 Guarantees Cell-Cycle Elongation at the *Xenopus laevis* Mid-blastula Transition

Clara Collart, James C. Smith, and Philip Zegerman

Figure S1

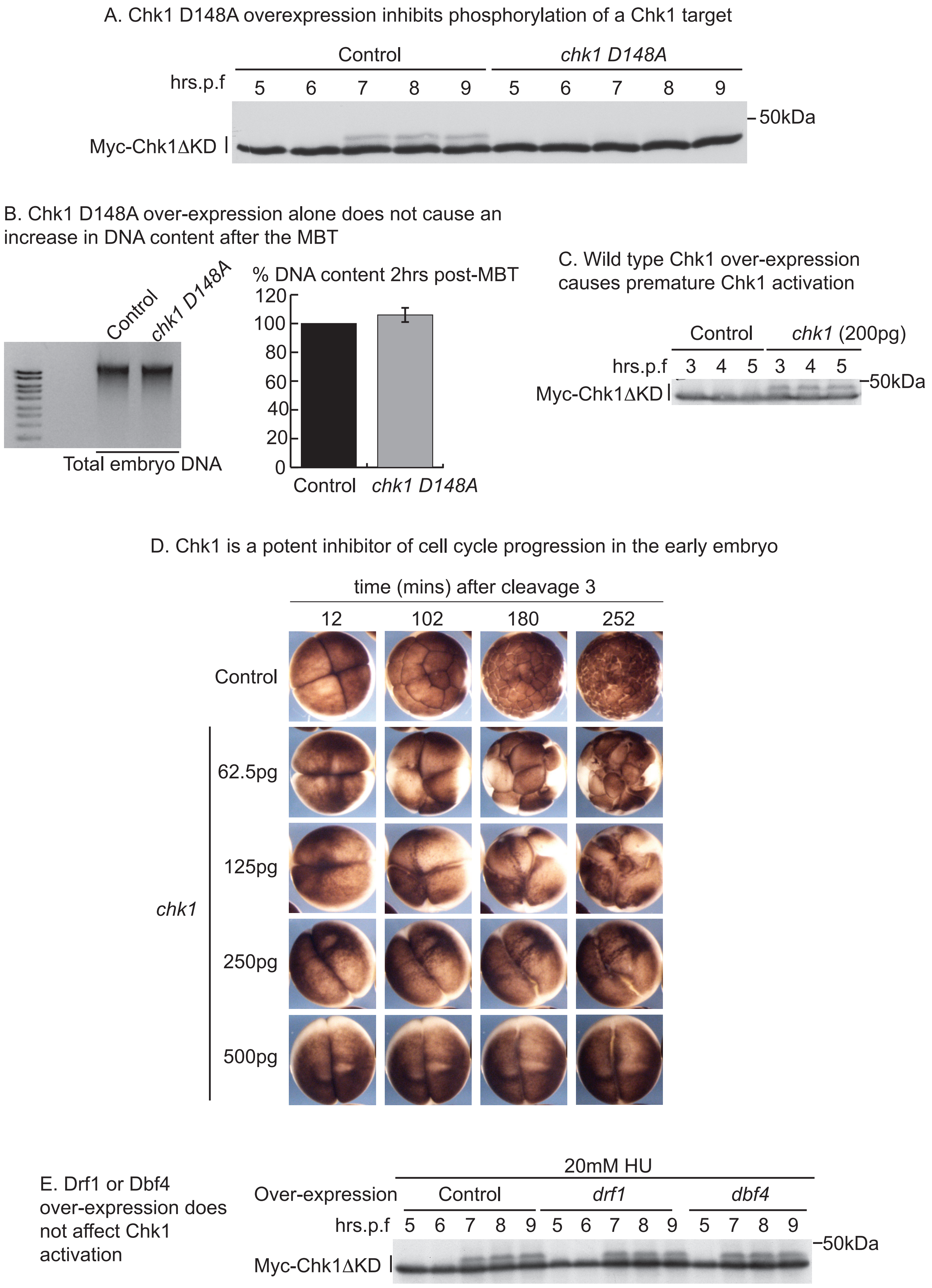

A. Chk1 D148A overexpression inhibits phosphorylation of a Chk1 target
Control
chk1 D148A
hrs.p.f
5 6 7 8 9 5 6 7 8 9
50kDa
Myc-Chk1ΔKD
B. Chk1 D148A over-expression alone does not cause an increase in DNA content after the MBT
Control
chk1 D148A
Total embryo DNA
% DNA content 2hrs post-MBT
120
100
80
60
40
20
0
Control
chk1 D148A
C. Wild type Chk1 over-expression causes premature Chk1 activation
Control
chk1 (200pg)
hrs.p.f
3 4 5 3 4 5
50kDa
Myc-Chk1ΔKD
D. Chk1 is a potent inhibitor of cell cycle progression in the early embryo
time (mins) after cleavage 3
12
102
180
252
Control
chk1
62.5pg
125pg
250pg
500pg
E. Drf1 or Dbf4 over-expression does not affect Chk1 activation
20mM HU
Over-expression
Control
drf1
dbf4
hrs.p.f
5 6 7 8 9 5 6 7 8 9 5 7 8 9
50kDa
Myc-Chk1ΔKD

Figure S2.
Only over-expression of *treslin*, *recq4* and *cut5* together with the *chk1 D148A* mutant permits fast MBT cycles

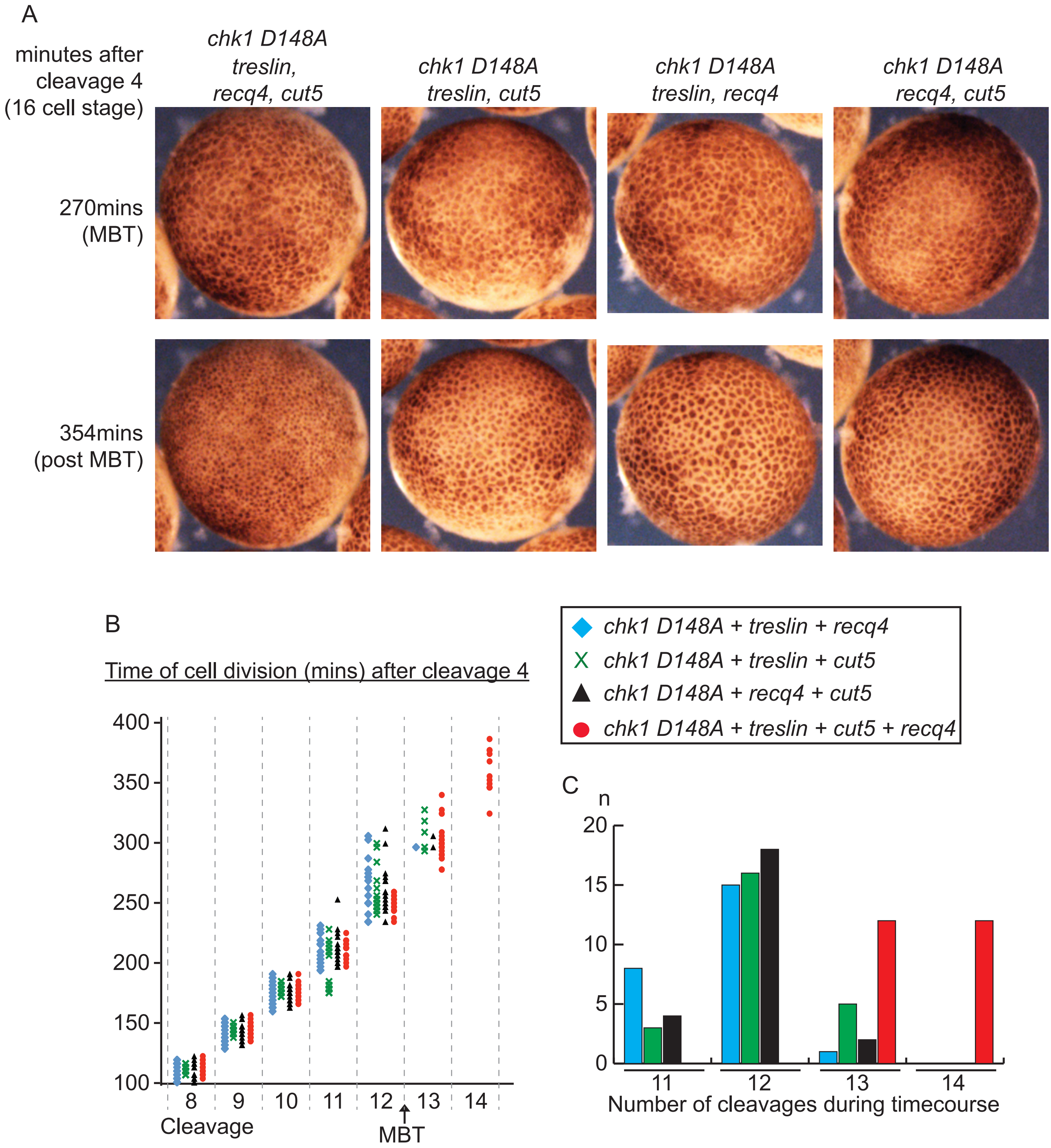

Figure S3.
Inhibition of DNA replication by injection of aphidicolin is sufficient to block the cell division, pre-MBT.

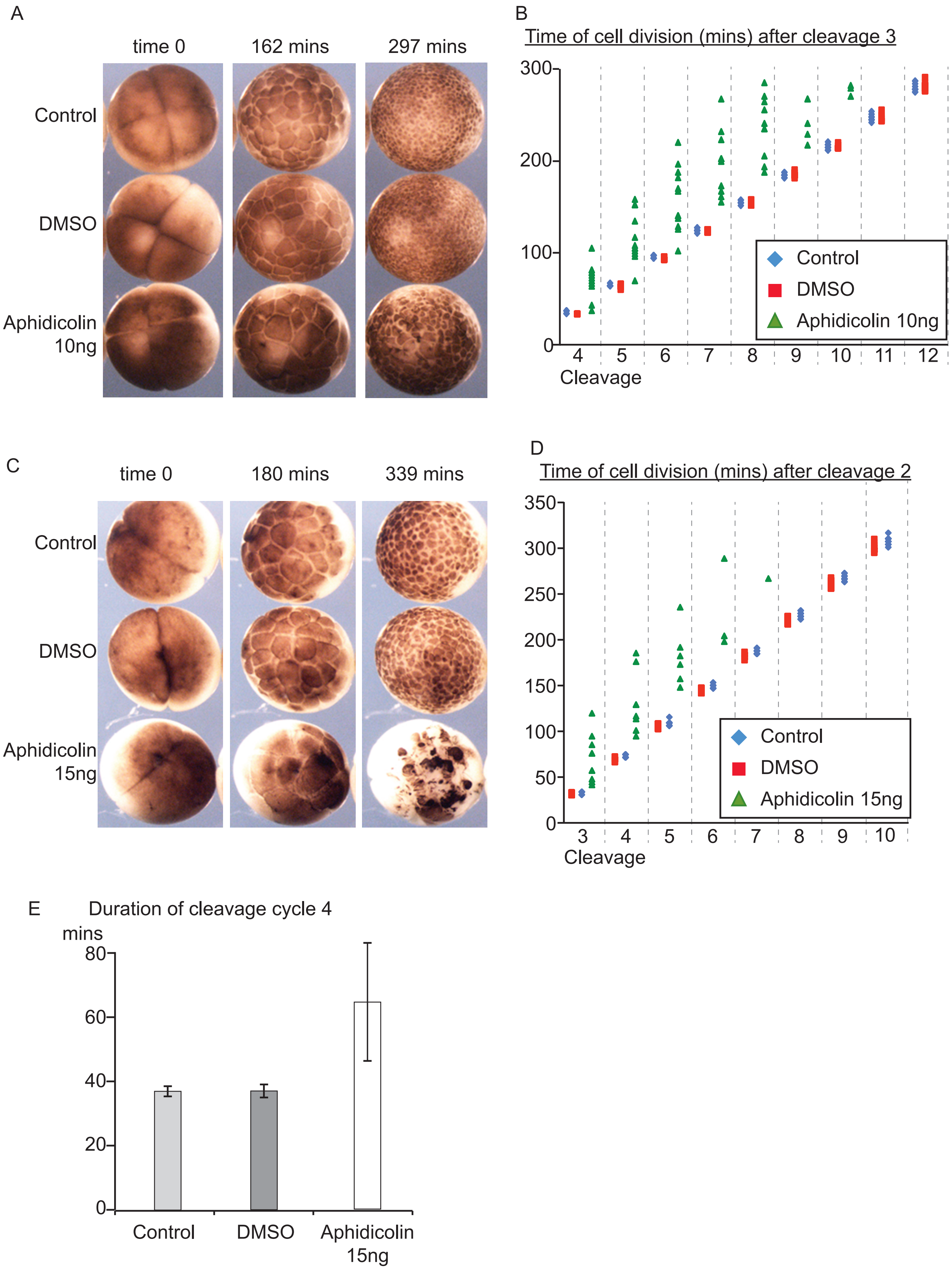

Figure S4.
Over-expression of *cdk1-AF* does not prevent Chk1-mediated elongation of pre-MBT cycles

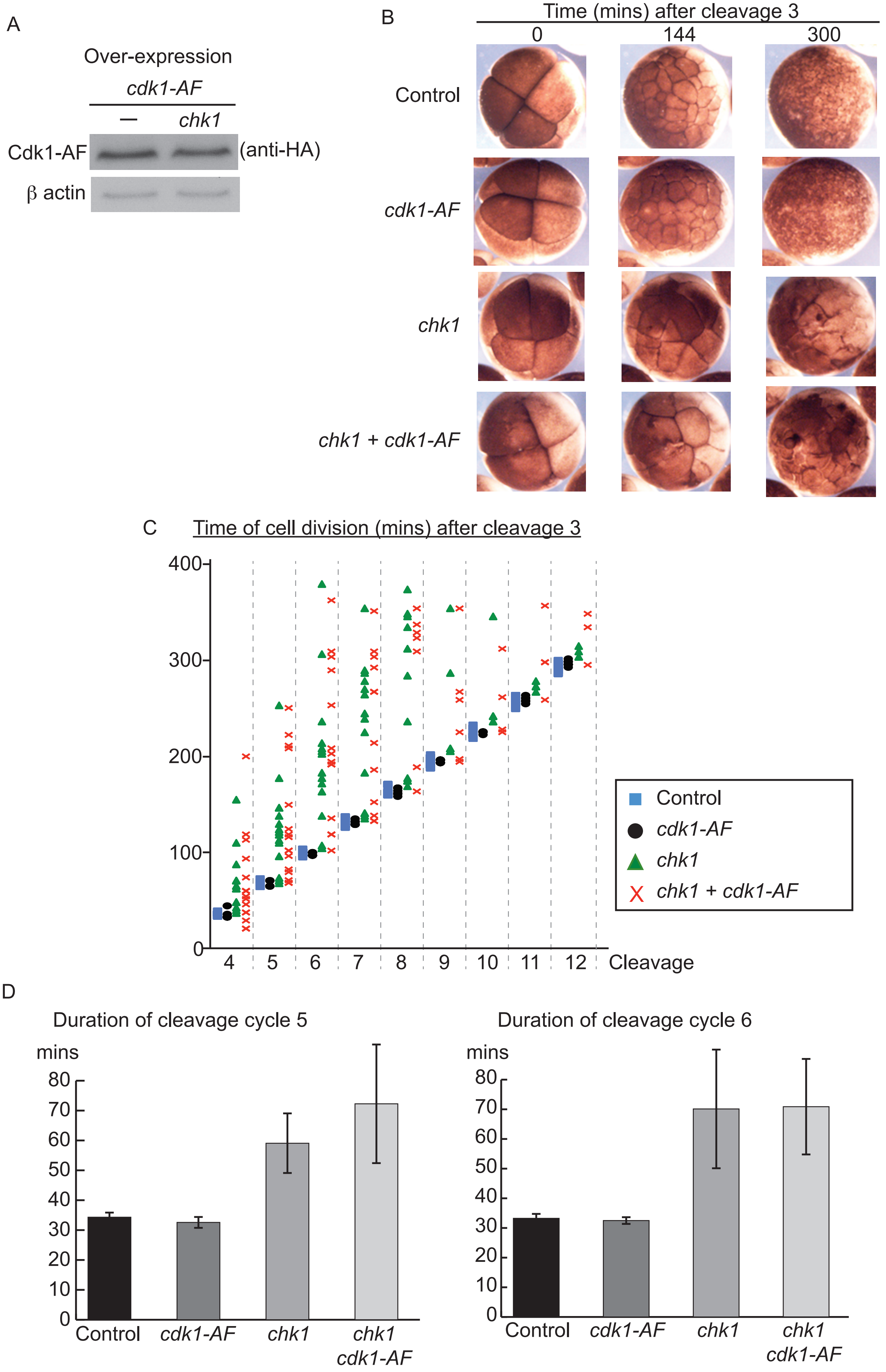

Figure S5

Over-expression of *cdk1-AF* does not affect the elongation of the cell cycle at the MBT

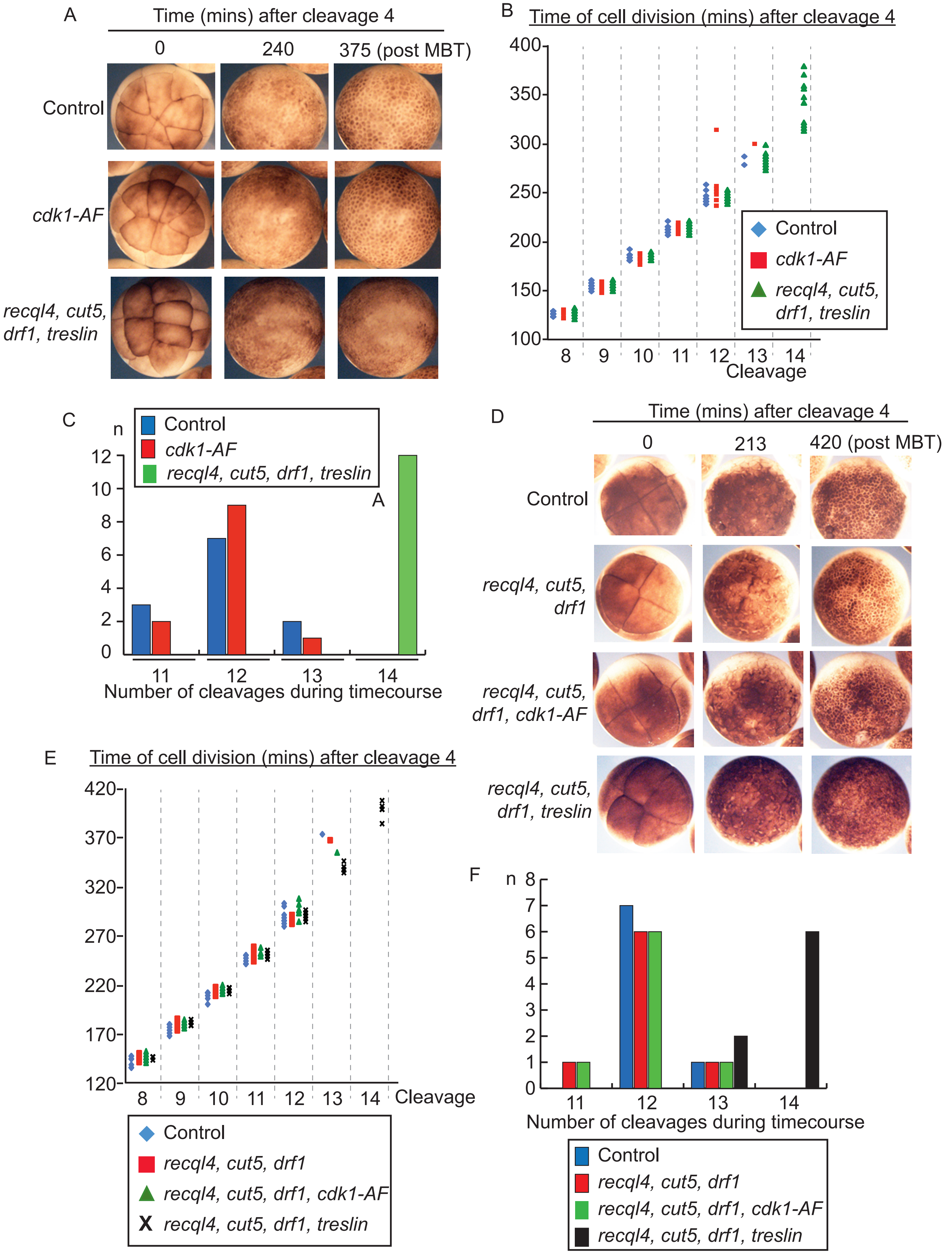

Figure S6
Over-expression of β *trcp* causes earlier Drf1 degradation and premature cell cycle elongation

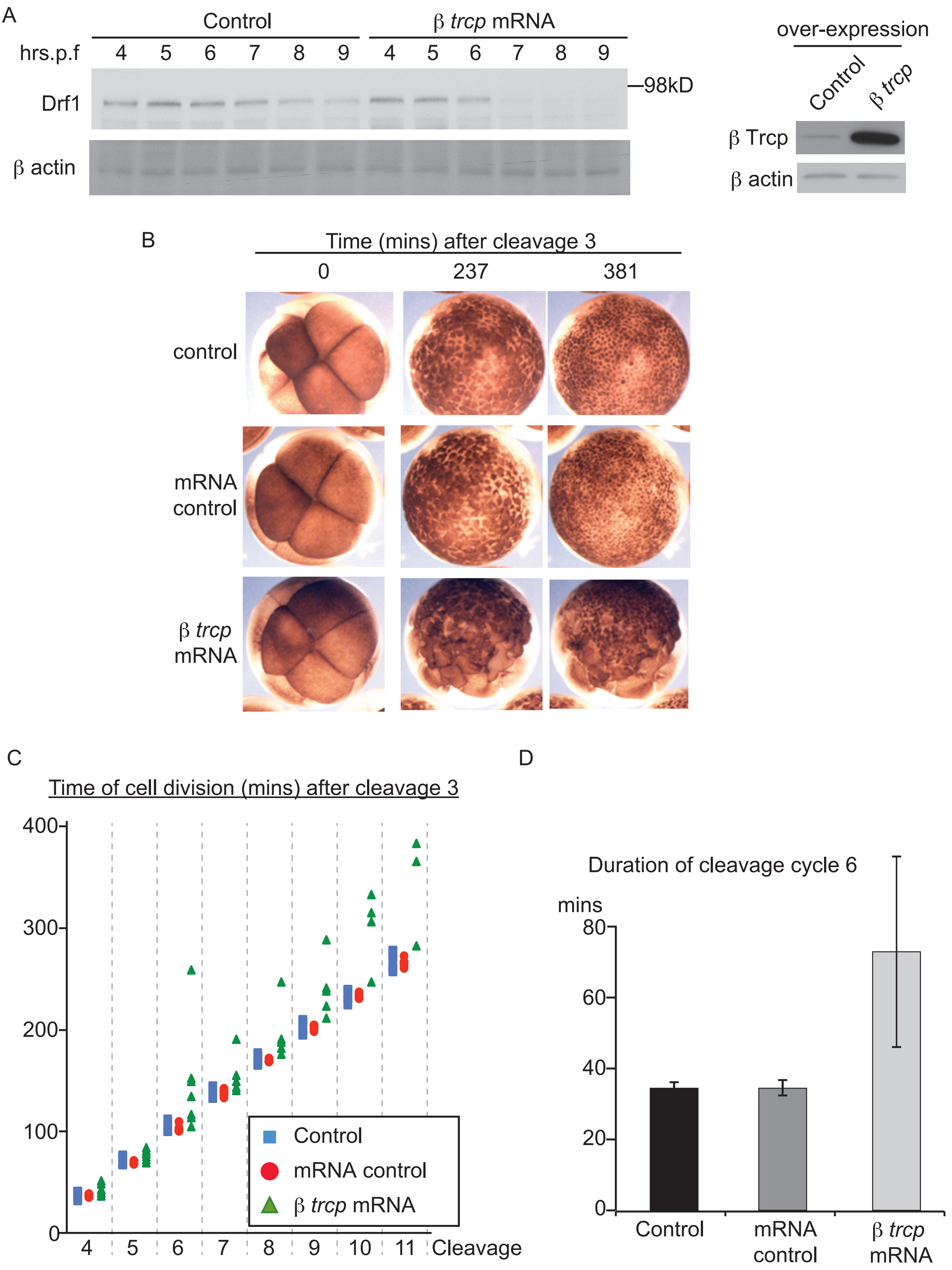

Figure S7

Unlike over-expression, stabilisation of Drf1 by down-regulation of β-*trcp* is not sufficient to allow rapid, synchronous divisions at the MBT.

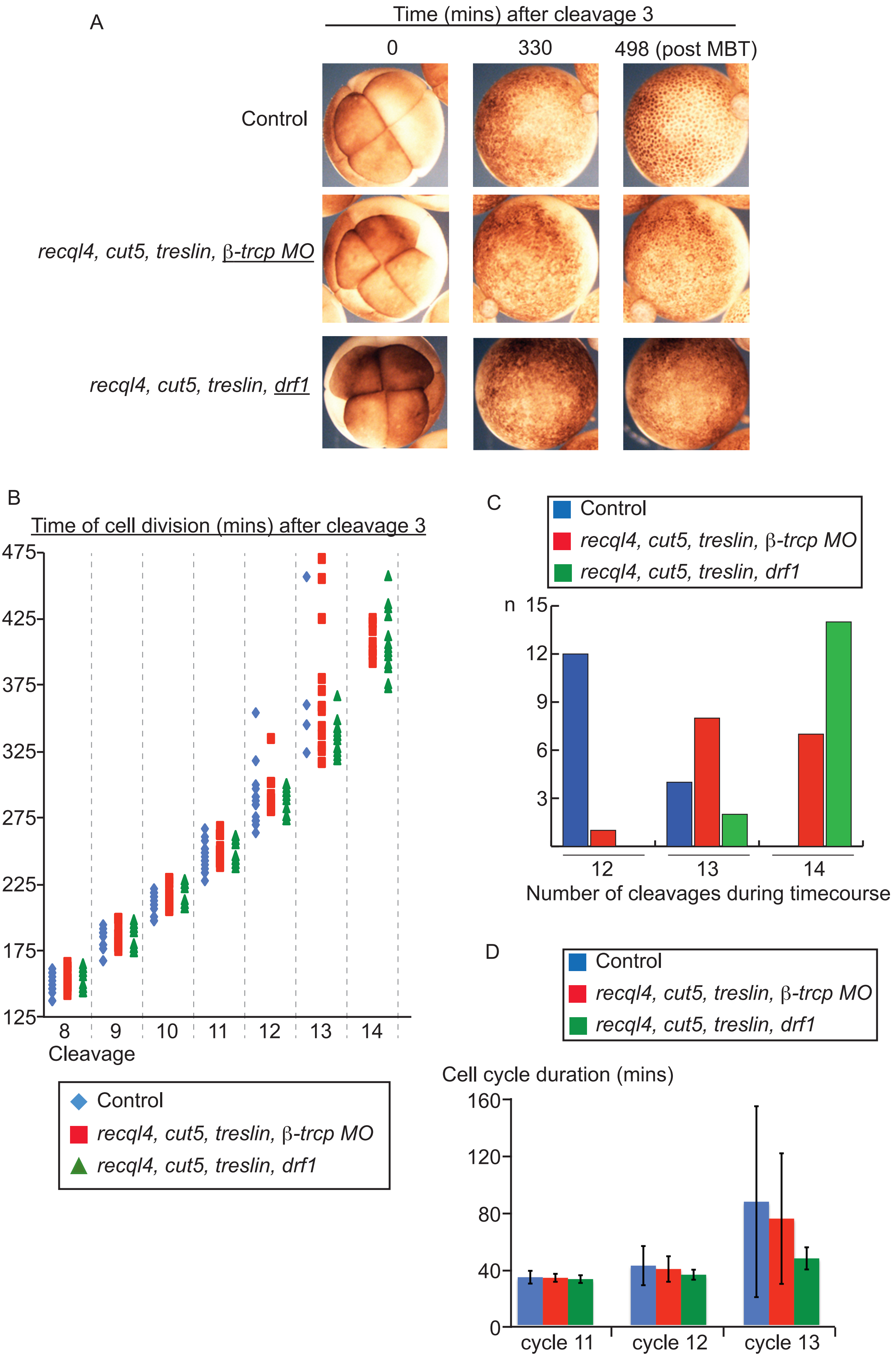

Supplementary figure legends

Figure S1. Chk1 inactivation blocks phosphoryation of downstream targets and Chk1 over-expression inhibits cell cycle progression in the early embryo. Related to Figure 1 and 3.

A. A Myc tagged fragment of Chk1 lacking the kinase domain (Chk1ΔKD) is phosphorylated by endogenous Chk1, resulting in a mobility shift in SDS-PAGE. This is an anti-myc Western blot of Chk1ΔKD from staged embryos at the indicated number of hours post fertilisation (hrs.p.f). Embryos were injected at the one cell stage either with water (control) or with mRNA corresponding to the *chk1* dominant negative mutant (D148A).

B. The DNA content of embryos 2hrs post MBT, injected at the one-cell stage with either water or *chk1 D148A* mRNA, was quantified on agarose gels (left) using ImageJ. The DNA content of 3 embryos was averaged and the control embryo DNA content set to 1 (right). Data are represented as mean ± SD

C. As in A except wild type *chk1* mRNA (200pg) was injected.

D. Images of embryos injected at the 1-cell stage either with water (control) or with increasing amounts of wild type *chk1* mRNA. Images are from time-lapse movies of these embryos at the indicated times after cleavage 3 (8 cell embryo).

E. As in A except embryos were incubated with 20mM HU with or without injection of mRNA for *drf1* or *dbf4.*

Figure S2. Over-expression of Treslin, Recq4 and Cut5 is required for rapid cell divisions at the MBT in embryos expressing Chk1 D148A. Related to Figure 2.

A. Embryos were injected in both blastomeres at the 2-cell stage with the indicated mRNA or water (control) and followed by time-lapse imaging. The 4th division, generating the 16-cell embryo was set to time zero. See also Movie S3.

B. The division of individual cells from A were followed throughout the movie. Each time-point represents the division of a single cell. Cleavages 4-7 are excluded for simplicity. n=24 cells from 6 embryos for each condition.

C. Total number of divisions each cell in B undergoes until the end of the time-lapse movie. The colour code is the same for both B and C.

Figure S3. Injection of aphidicolin is sufficient to block cell division before the MBT. Related to Figure 3.

A. Water (control), DMSO or aphidicolin was injected in both blastomeres at the 2-cell stage and followed by time-lapse imaging. The 3rd division, generating the 8-cell embryo was set to time zero.

B. The division of individual cells from A were followed throughout the movie. Each time-point represents the division of a single cell. n=12 cells from 3 embryos for each condition.

C,D as A,B.

E. Quantitation of the average duration of cell cycle 4 from C,D. n=12 cells from 3 embryos for each condition. Data are represented as mean ± SD, which indicates the synchrony of cell divisions.

Figure S4. Over-expression of Cdk1-AF does not prevent premature cell cycle lengthening after over-expression of Chk1. Related to Figure 3.

A. Anti HA western blot of N-terminally HA tagged Cdk1-AF from embryos 3 hrs.p.f.

B. Water (control), *chk1* mRNA, *cdk1-AF* mRNA or both mRNAs were injected in both blastomeres at the 2-cell stage and followed by time-lapse imaging. Total mRNA injected for *chk1* was 50pg, while for *cdk1-AF* it was 500pg. The 3rd division, generating the 8-cell embryo was set to time zero.

C. The division of individual cells from B were followed throughout the movies. Each time-point represents the division of a single cell. n=16 cells from 4 embryos for each condition.

D. Quantitation of the average duration of cell cycle 5 (left) and cycle 6 (right) from C. Data are represented as mean ± SD, which indicates the synchrony of cell divisions.

Figure S5. Over-expression of Cdk1-AF does not affect the duration of the cell cycle at the MBT. Related to Figure 3.

A. Embryos were injected in both blastomeres at the 2-cell stage with *cdk1-AF* mRNA (1ng total), the four limiting replication factors (300pg each total) or water (control) and followed by time-lapse imaging. The 4th division, generating the 16-cell embryo was set to time zero.

B. The division of individual cells from A were followed throughout the movies. Each time-point represents the division of a single cell. n=12 cells from 3 embryos for each condition.

C. Total number of divisions each cell in B undergoes until the end of the time-lapse movie. The colour code is the same for both B and C.

D. Embryos were injected in both blastomeres at the 2-cell stage with mRNAs or water (control) as indicated and followed by time-lapse imaging. The amounts of injected mRNAs were 500ng total for *cdk1-AF* and 300pg each total for *drf1, recq4*, *cut5* and *treslin*. The 4th division, generating the 16-cell embryo was set to time zero.

E. The division of individual cells from D were followed throughout the movies. Each time-point represents the division of a single cell. n=8 cells from 2 embryos for each condition.

F. Total number of divisions each cell in E undergoes until the end of the time-lapse movie. The colour code is the same for both E and F.

Figure S6. Over-expression of β-Trcp causes premature cell cycle elongation. Related to Figure 4.

A. (right) Anti β-Trcp western blot from stage 7 embryos (just before MBT) after injection at the 1 cell stage with water (control) or 1ng of *β-trcp* mRNA. (left) Anti-Drf1 western blot from staged embryos.

B. Water (control), antisense *β-trcp* mRNA (mRNA control) or *β-trcp* mRNA were injected in both blastomeres at the 2-cell stage and followed by time-lapse imaging. The total amount of injected mRNA was 5ng. The 3rd division, generating the 8-cell embryo was set to time zero.

C. The division of individual cells from B were followed throughout the movies. Each time-point represents the division of a single cell. n=8 cells from 2 embryos for each condition.

D. Quantitation of the average duration of cell cycle 6 from C. Data are represented as mean ± SD, which indicates the synchrony of cell divisions.

Figure S7. Over-expression of Drf1 is required to allow rapid, synchronous divisions at the MBT. Related to Figure 7D

A. Embryos were injected in both blastomeres at the 2-cell stage with mRNAs, anti-*β-trcp* morpholinos (MO) or water (control) as indicated and followed by time-lapse imaging. The total amounts of injected mRNAs/MO were *drf1, recq4*, *cut5* and *treslin* (300pg each), *β-trcp* morpholinos (80ng). The 3rd division, generating the 8-cell embryo was set to time zero.

B. The division of individual cells from A were followed throughout the movies. Each time-point represents the division of a single cell. n=16 cells from 4 embryos for each condition.

C. Total number of divisions each cell in B undergoes until the end of the time-lapse movie. The colour code is the same for B-D.

D. Quantitation of the average duration of cell cycles 11-13 from B. Data are represented as mean ± SD, which indicates the synchrony of cell divisions. Notably only over-expression of Drf1 (together with the other 3 limiting factors) allows for rapid and synchronous cleavages in cycles 12/13.
